# Comprehensive analysis of genomic and immunological profiles in Chinese and Western hepatocellular carcinoma populations

**DOI:** 10.18632/aging.202853

**Published:** 2021-04-18

**Authors:** Wei Li, Hong Wu, Xuewen Xu, Yange Zhang

**Affiliations:** 1 Department of Plastic and Burns Surgery, West China Hospital, Sichuan University, Chengdu 610041, China; 2 Department of Liver Surgery and Liver Transplantation, State Key Laboratory of Biotherapy and Cancer Center, West China Hospital, Sichuan University and Collaborative Innovation Center of Biotherapy, Chengdu 610041, China

**Keywords:** hepatocellular carcinoma, heterogeneity, whole exome sequencing, tumor-infiltrating immune cell, TP53

## Abstract

In this study, we explored the genomic and immune cell infiltration profiles among four distinct Hepatocellular carcinoma (HCC) types. This study included 100 patients (all tumors and adjacent liver tissues received WES sequencing) with HCC from the West China Hospital (WCH) and patients were divided into WCH-HBV-HCC group and WCH-NonHBV-HCC group. Additionally, this study included 106 HBV-related HCC (TCGA-HBV-HCC) and 69 alcoholic HCC (TCGA-Alcol-HCC) patients from the TCGA. We analyzed the high-frequency gene mutation, copy number variation (CNV), mutation spectrum, signatures and immune cell infiltration of these four groups. This study showed significant differences in gene mutation and CNV level among four HCC groups. Compared to genomic level, there is no significant difference between TCGA-HBV-HCC and TCGA-Alcol-HCC groups in fractions of tumor-infiltrating immune cells. According to the status of immune cell infiltration, patients were classified into immune-HIGH, immune-MIX and immune-LOW group, respectively. In the WCH-HBV-HCC and TCGA-HBV-HCC groups, more patients in the Immune-LOW group had TP53 mutation. Except for TP53, neither the other gene mutation nor tumor mutation burden was found to be associated with immune cell infiltration in the WCH-HBV-HCC, TCGA-HBV-HCC and TCGA-Alcol-HCC groups. In the CNV level, we found that samples with low immune infiltrate had higher number of deleted or amplified genes in the TCGA-HBV-HCC and TCGA-Alcol-HCC groups. We found comprehensive genomic heterogeneity among four HCC groups. The total gene CNV level, not the mutational burden of HCC, is associated with immune cell infiltration in HCC. TP53 mutation may injury the immune response of the HBV-related HCC.

## INTRODUCTION

Hepatocellular carcinoma (HCC) accounts for more than 90% of primary liver cancer. Compared with other types of tumors, the risk factors of HCC are well defined [1]. HCC often occurs in livers with chronic liver disease and significant cirrhosis [2, 3]. Factors related to cirrhosis include Hepatitis B virus (HBV) infection, Hepatitis C virus (HCV) infection, alcohol abuse and non-alcoholic steatohepatitis (NASH), etc. [4, 5]. HBV is one of the main risk factors for HCC. Owing to the high infection rate of HBV, Asia and Sub-Saharan Africa have the highest incidence of HCC [2]. There are a large number of patients with HBV infection in China, and in parallel, HBV-related HCC accounts for nearly 85% of all newly-diagnosed HCC [6]. In Africa, HCC occurrence results from a complex interplay between aflatoxin B1 and HBV infection [1]. In North America, Europe and Japan, HCV is one of the most common risk factors for HCC [7]. In addition to the above causes, other risk factors for HCC include metabolic syndrome (characterized by diabetes and obesity) and adenovirus infection, etc. [7].

HCC is biologically heterogeneous [8]. HCC that develops on the background of cirrhosis often derives from malignant transformation of dysplastic nodules [3, 9]. In patients without cirrhosis, HCC can occur directly in livers with only HBV infection or NASH [3]. In addition, HCC can derive from the malignant transformation of hepatic adenoma [3]. These types of HCC have different molecular mutation profile. Previous studies regarding genomics and transcriptomics of HCC have identified many driver mutations and alterations in expression profiles of HCC. For example, research based on The Cancer Genome Atlas (TCGA) suggests that TP53, CTNNB1 and TERT promoters are the most common mutations in HCC from the Western populations [10]. However, given the heterogeneity of HCC, studies that focus on Western HCC cases cannot reveal the molecular characteristics and individual heterogeneity of HCC in China. Chinese HCC patients may have an extensive heterogeneity in comparison to HCC in the European and American countries in terms of pathogenesis, epidemiological features, biological behavior, staging, and treatment strategies [10–12]. Additionally, with the changes in diet and environment, the pathogenic factors of HCC in China may also undergo certain changes. For example, alcoholic hepatitis and the metabolic syndrome may gradually become the main risk factor for HCC occurrence [13]. Consequently, researches exploring the heterogeneity of HCC need to be continued and updated.

It is significant to illustrate the tumor heterogeneity for HCC treatment. After Sorafenib, a series of targeted therapeutic drugs for HCC were launched in clinical trials [14, 15]. However, for patients with advanced HCC, there are no targeted drugs that can achieve obvious efficacy and can be widely used clinically [16]. The main challenges faced by targeted drugs such as Sorafenib are low response rate and primary or secondary drug resistance [17]. In addition to molecular targeted therapy, immunotherapy, especially immune checkpoint inhibitors (such as anti-PD-1, PD-L1, LAG3, TIM3, Tregs and immunosuppressive factors released by Tregs such as TGF-β, etc.), are utilized for HCC treatment [18]. However, the response rate of immunotherapy in HCC is also low, and there is no biomarker that can accurately predict the effect of immunotherapy [19]. In summary, it is a necessity for individualized treatment to explore the heterogeneity of HCC at the molecular level and screen out patients sensitive to targeted therapy and immunotherapy.

This study includes HBV-related HCC and alcohol-related HCC patients in the Western populations (HBV and alcoholic HCC are two most common types of HCC in the TCGA database). In addition, this study also includes cases with HBV-related HCC and non-HBV/non-HCV related HCC patients in China. Based on genome sequencing, the present study will compare the genomic heterogeneity of the above four patient populations and find new potential therapeutic targets. In this study, we will analyze the infiltration of immune cells in HCC tumors and systematically compare the heterogeneity of the immune microenvironment of the above-mentioned different HCC populations. The interaction between mutational profiles and the host immune status in HCC has been less well investigated. In this study, we classified the HCC immune microenvironment according to the number of tumor-infiltrating immune cells, and analyzed whether the immune cell infiltration of HCC is related to specific gene mutations, tumor mutation burden (TMB) or CNV status.

## MATERIALS AND METHODS

### Sample collection

This study includes 100 HCC patients diagnosed from June 2009 to December 2014 in the West China Hospital, Sichuan University. The tissue samples in this study include tumor tissue and matched liver tissue adjacent to the tumor. The application and use of samples were reviewed and approved by the Institutional Ethics Committee of the West China Hospital. In addition, this study include 175 patients from the TCGA (http://cancergenome.nih.gov/) database. HBV-related HCC from the West China Hospital was abbreviated as WCH-HBV-HCC; HBV-related HCC in the Western population of TCGA database was abbreviated as TCGA-HBV-HCC; NonHBV-related HCC in the West China Hospital was abbreviated as WCH-NonHBV-HCC; NonHBV-related HCC in the Western population from the TCGA database was only included alcoholic HCC, abbreviated as TCGA-Alcol-HCC. All HBV-related HCC patients were positive for hepatitis B surface antigen (HBsAg). The main causes of 19 nonHBV-related HCC patients in the West China Hospital are as follows: 3 metabolic syndrome related diseases (diabetes and obesity), 2 alcoholic, and the remaining 14 patients have no obvious cirrhosis and no clear cause. Tumor staging is based on the eighth edition of the American Cancer Society (AJCC)-TNM staging. The degree of tumor differentiation according to Edmondson-Steiner standard is divided into I-IV grade. I-II grade is defined as high differentiation and III-IV grade is defined as low differentiation.

### Immunohistochemical staining

The tissue microarray is constructed by the Xinchao Biotechnology (Shanghai, China). The steps of the immunohistochemical (IHC) staining were described previously [20]. Details of the primary antibodies used in IHC staining is shown in the Supplementary Table 1.

In the WCH-HBV-HCC and WCH-NonHBV-HCC groups, the number of three types of immune cells were evaluated by IHC staining including pan-T cells (CD3), B cells (CD20) and cytotoxic T cells (CD8). Before counting immune cells, five representative areas were selected (each area is 1mm^2^) at 100 × magnification, and then the number of positive immune cells was counted at 200 × magnification. The average of the five areas is the number of positive immune cells. The values of other immune markers were recorded as cells/mm^2^. Methods for analysis of tumor-infiltrating immune cells for samples from the TCGA-HBV-HCC and TCGA-Alcol-HCC groups was shown in the Supplementary Materials and Methods.

### Whole exome sequencing

After DNA extraction (UPure Tissue DNA Kit, BioBase, Sichuan, CN), we performed DNA quality control in three aspects: (1) Evaluation of DNA degradation by agarose gel electrophoresis; (2) Agilent Bioanalyzer 2100 (Agilent, USA) detects the size of DNA sample fragments; (3) Thermo Scientific™ NanoDrop™ (Thermo Fisher Scientific, USA) tests the purity of DNA samples (OD260/280 and OD230/260).

The DNA was fragmented using the ultrasonic interrupter Bioruptor® (Diagenode, Belgium). After connecting the sequencing adaptor, the Illumina sequencing library was constructed utilizing Nextera DNA Library Prep Kit (Illumina, USA). For exon capture, Agilent Sureselect™ Human All Exon V6 kit (Agilent, USA) was used for liquid phase hybrid capture of the exon portion of DNA. After building the high-throughput sequencing library, the sequencing was performed on the platform Illumina Novaseq™ 6000 (Illumina, USA) with the sequencing mode of PE150. After removing the interference from PCR duplicates, the average sequencing depth of cancer tissues was 200 ×, and the average sequencing depth of adjacent tissues was 100 ×.

### Genomic analysis

We firstly used the FASTQ software to remove the joint sequences and low-quality sequences in the original data to obtain the "clean data" in the format of FASTQ. The BWA software was used to perform reference genome alignment (hg19 version) with the reads contained in paired FASTQ files, and the Picard software was utilized to identify and mark duplicate reads from BAM file. Next, GATK4.0 software was used to identify the mutations of SNP and Indel. In this study, when copy number variation (CNV) was detected, the sequencing depth was firstly corrected based on the GC content of the sequence, and finally the presence of CNV was determined after normalization of the data by clustering analysis. Tools used for CNV analysis includes GISTIC 2.0 [21] and CNVkit [22]. The significance of gene mutation was identified by MutsigCV [23] and visualized by maftools and ggplot2. The non-negative matrix factorization (NMF) method was based on the previous study [24]. The identified signatures were compared with the COSMIC signatures.

### Analysis of data from TCGA

The TCGA genomic and transcriptome sequencing data was obtained from the Genomic Data Commons (GDC) Data Portal (https://portal.gdc.cancer.gov/). We utilized the same analytic methods of genomic analysis for patients in the WCH and TCGA cohorts. The analytic methods of tumor-infiltrating immune cells in the TCGA-HBV-HCC and TCGA-Alcol-HCC cohorts were shown in the Supplementary Materials and Methods in detail.

### Statistical analysis

Categorical variables were expressed as number (%), and the statistical test method was Chi-square test or Fisher^,^ exact test. Continuous variables were expressed as mean ± standard deviation, and the statistical method was T-test or Kruskal-Wallis test. The FDR-corrected q values were used to compare the differences in frequency of the common gene mutation or CNV number between two groups, and the calculation method was the Benjamini-Hochberg method. Data analysis was carried out by R software 3.4.3.

## RESULTS

### Sample and clinical information

The clinicopathologic information of HCC patients from the West China Hospital is shown in Supplementary Table 2. Compared to patients in the WCH-HBV-HCC group, patients in the WCH-NonHBV-HCC group were older (P = 0.002), while the other clinicopathologic features were not significantly different between the two groups (all P values > 0.05). For patients in the TCGA group, patients with alcoholic HCC (TCGA-Alcol-HCC group) were older than those with HBV-related HCC (TCGA-HBV-HCC group) (P <0.001). In addition, compared to patients from the TCGA-HBV-HCC group, cases in the TCGA-Alcol-HCC group had higher proportion of white people (P <0.001), later TNM stage (P <0.001), better tumor differentiation (P <0.001), and lower proportion of microvascular and macrovascular invasion (P = 0.032). The remaining characteristics had no significant difference between the two groups (all P values > 0.05) (Supplementary Table 3).

### Somatic copy number variation

In this study, GISTIC 2.0 was used to identify copy number changes for patients in the HBV and non-HBV groups, including copy number amplification and deletion. We found that, there was a significant difference in total CNV levels for patients in the WCH-HBV-HCC group (Supplementary Figure 1B) and WCH-nonHBV-HCC group (Supplementary Figure 1C).

**Figure 1:**
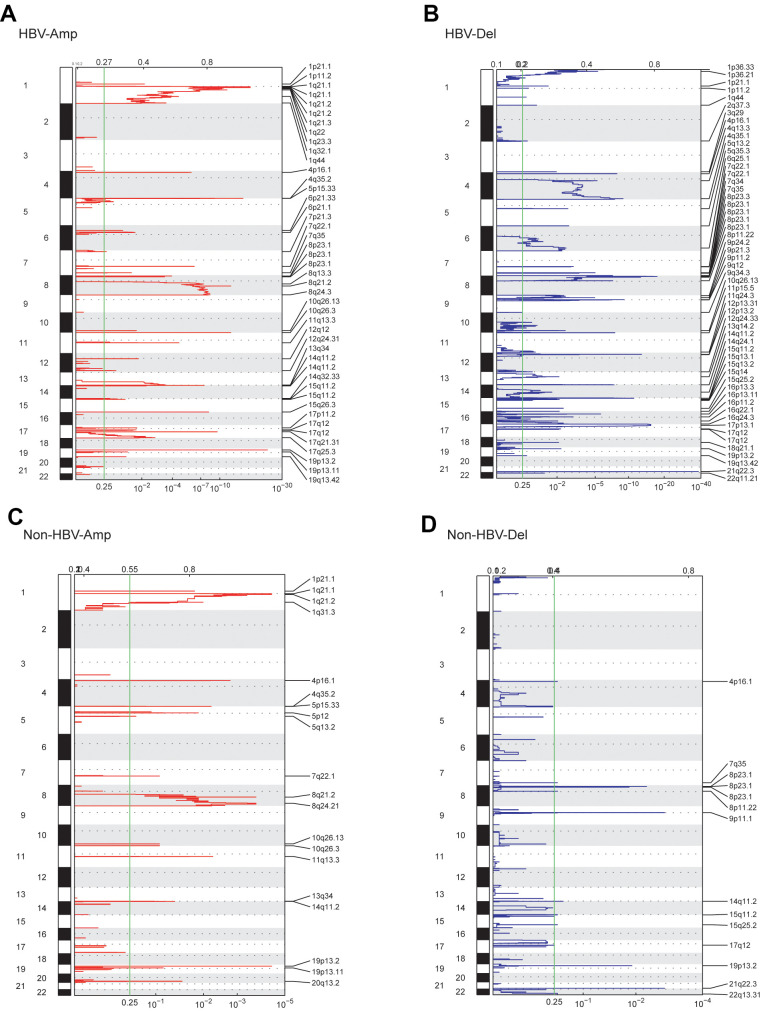
**GISTIC analysis showed the whole-genome distribution of copy number alterations.** (**A**) CNV amplifications in the WCH-HBV-HCC group. (**B**) CNV deletions in the WCH-HBV-HCC group. (**C**) CNV amplifications in the WCH-NonHBV-HCC group. (**D**) CNV deletions in the WCH-NonHBV-HCC group. GISTIC q-values (x-axis) for deletions (**B**, **D**) and amplifications (**A**, **C**) are plotted across the genome (y-axis). The green vertical line is where the q-value is 0.25.

For patients in the WCH-HBV-HCC group, copy number amplification (CNV-Amp) was found mainly on chromosomes 1 and 8, and other locations including 4p, 4q, 5p, 6p, 7p, 7q, 11q, 12q, 13q, 14q, 15q, 17p, 17q, 19p and 19q, etc. Copy number deletion (CNV-Del) was observed predominantly on chromosomes 7q, 8p, 15q, and other locations including 1p, 1q, 2q, 3q, 4p, 4q, 5q, 6q, 9p, 9q, 10q, 11p, 11q, 16p and 17q, etc. Among WCH-NonHBV-HCC patients, CNV-Amp was found to mainly locate at 1q, 8q, 10q, 11p, and 19p; CNV-Del was found to mainly locate at 8p, 15q, and 9p (Figure 1). In summary, the CNV levels in the WCH-HBV-HCC group and WCH-NonHBV-HCC group are significantly distinct.

Firstly, we compared the CNV levels of patients in the WCH-HBV-HCC group and the TCGA-HBV-HCC group. We found that in the above two groups, the locations and frequencies of CNV-Amp and CNV-Del were obviously different between two groups (Supplementary Figure 2A, 2B). For example, cases in the WCH-HBV-HCC group had CNV-Amp in 4p, 4q, 5p, 6p and 14q and CNV-Del in 14q, 15q, 16p and 21q, while CNV in these locations were not significantly observed in the TCGA-HBV-HCC group. Besides, significant difference was also observed between the WCH-NonHBV-HCC group and the TCGA-Alcol-HCC group (Supplementary Figure 2C, 2D).

Additionally, patients in the WCH-HBV-HCC group and the TCGA-HBV-HCC group were compared at the gene level (genes affected by CNV). The number of unique CNV-Amp genes in the WCH-HBV-HCC group and TCGA-HBV-HCC group were 668 and 771, respectively, while the number of CNV-Amp genes shared by the two was 184. The number of unique CNV-Del genes of the above two groups is 2487 and 236, respectively, while the number of CNV-Del genes shared by the two groups is 684. Supplementary Figure 3 shows parts of the CNV-affected genes that are common or unique in the WCH-HBV-HCC and TCGA-HBV-HCC groups (Supplementary Figure 3A, 3B), or WCH-NonHBV-HCC and TCGA-Alcol-HCC groups (Supplementary Figure 3C, 3D). The shared CNV-Amp genes include CYC1, ATP4B, SCCPDH and KISS1, etc. The shared CNV-Del genes include ACADVL and FAM138A, etc. In addition to the above-mentioned shared CNV genes, there are a large number of CNV-affected genes that only observed in their respective cohorts. For example, CNV-Amp of MUC16, ZDHHC1, DUX4, NBPF20, PPIAL4C, REXO1L2P, RASA4 and CCND1 was only found in the WCH-HBV-HCC group, and CNV-Amp of ADCY8, TPCN2, FOXK2 and CAPZA2 was only shown in the TCGA-HBV-HCC group. In addition, in line with expectations, due to differences in pathogenic causes, the locations of CNV (Supplementary Figure 2C, 2D) and the genes affected by CNV (Supplementary Figure 3C, 3D) for patients in the WCH-NonHBV-HCC and TCGA-Alcol-HCC cohorts are significantly heterogeneous.

In addition to the genes shown in Supplementary Figure 3, there are a large number of common genes affected by CNV that appear separately in the WCH-HBV-HCC group and the TCGA-HBV-HCC group (Supplementary Figures 1–4). For example, CNV-Amp of genes such as FGF4, FGF19, ETV1, PMS2, RPTOR, CARD11, ERCC5 and RAC1 appeared only in the WCH-HBV-HCC group, and CNV-Amp of genes such as MET, MYC, AKT3, AGO2, BRD4 and IKBKE appeared only in the TCGA-HBV-HCC group. In addition, we found that CNV-Del of genes such as CDKN2A, AXIN1, ACVR1, PTPRD and ATM only appeared in the WCH-HBV-HCC group, while CNV-Del of genes such as FGFR2, STK11, TCF3, and KMT5A were only shown in the TCGA-HBV-HCC group.

### Somatic mutation and tumor mutation burden

**Figure 2:**
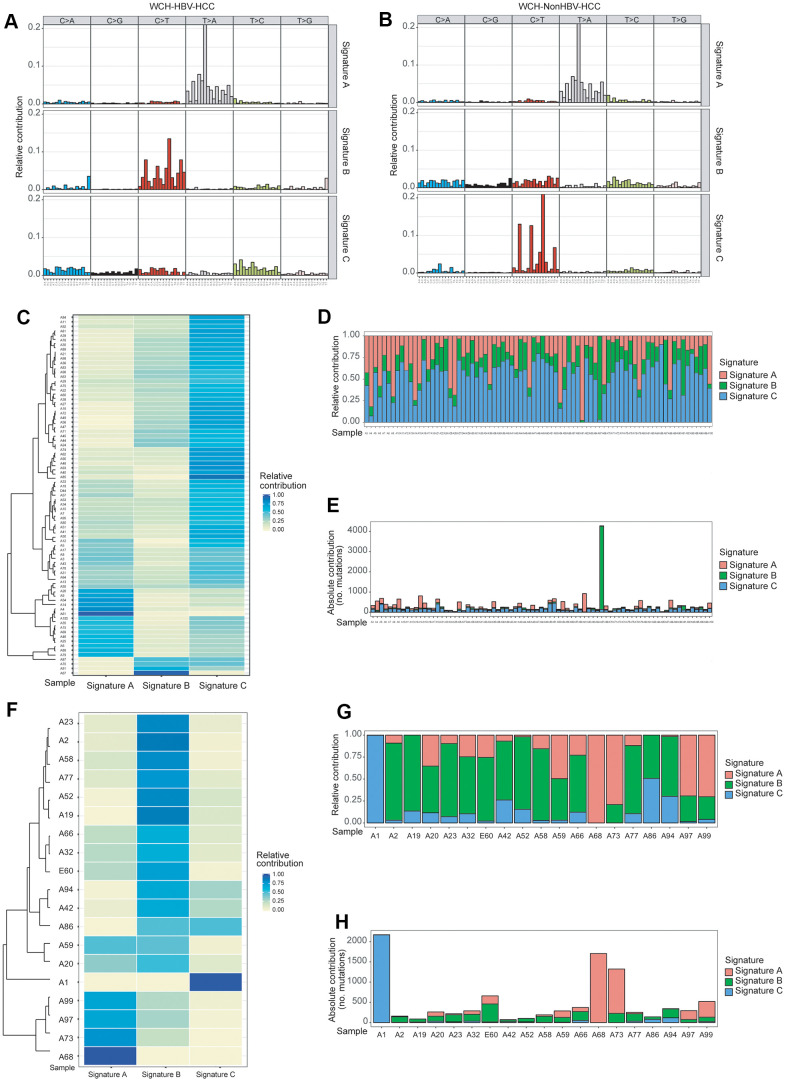
**Identification of mutation Signatures in the WCH group.** (**A**) Patterns of 3 signatures (Signatures **A**–**C**) identified in the WCH-HBV-HCC group. (**B**) Patterns of 3 signatures (Signatures **A**–**C**) identified in the WCH-NonHBV-HCC group. (**C**) The distribution of mutation Signatures that were identified in the WCH-HBV-HCC group. (**D**) The relative contribution of the 3 Signatures in samples from the WCH-HBV-HCC group. (**E**) The contributions of mutational signatures to tumors in the WCH-HBV-HCC group. The sample names are displayed on the horizontal axis, whereas the vertical axis depicts the number of mutations of samples in the WCH-HBV-HCC group. (**F**) The distribution of mutation Signatures that were identified in the WCH-NonHBV-HCC group. (**G**) The relative contribution of the 3 Signatures in samples from the WCH-NonHBV-HCC group. (**H**) The contributions of mutational signatures to tumors in the WCH-NonHBV-HCC group.

There are 4.5 (median) somatic mutations detected per million bases in WCH-HBV-HCC group, and the number is 5 (median) in WCH-NonHBV-HCC group. As shown in Figure 2, the most common mutational genes in patients from the WCH-HBV-HCC group are TP53, TTN, MUC4, CTNNB1, CDC27 and MUC16, etc. The most common mutations in patients from the WCH-NonHBV-HCC group are MUC16, TP53, TTN, ASTN1, ARID1A and HMCN1, etc. The most common mutant genes in cases from the TCGA-HBV-HCC group are TP53, CTNNB1, TTN, MUC16, AXIN1 and MUC4, etc. The most commonly mutated genes for patients in the TCGA-Alcol-HCC group are TP53, CTNNB1, TTN, PCLO, ALB and MUC16, etc. We found that more than half (51%) of patients in WCH-HBV-HCC cohort had TP53 mutations, while CTNNB1 mutations accounted for only 28% (ranking fourth). Other highly-mutated bur rarely-reported genes include TTN, MUC4, CDC27, MUC16, PCLO and UBA52, etc. The most frequently mutated genes in WCH-NonHBV-HCC patients are MUC16 and TP53, and only 4 patients in this group had CTNNB1 mutation. In contrast, in the TCGA-HBV-HCC group and the TCGA-Alcol-HCC groups, TP53 and CTNNB1 mutations are the most common genetic mutations. The sum of patients with TP53 or CTNNB1 mutation exceeds half of the individual cohort. Most of the samples do not have TP53 and CTNNB1 mutations simultaneously, which indicates that these two mutations represent two distinct types of tumors.

**Figure 3:**
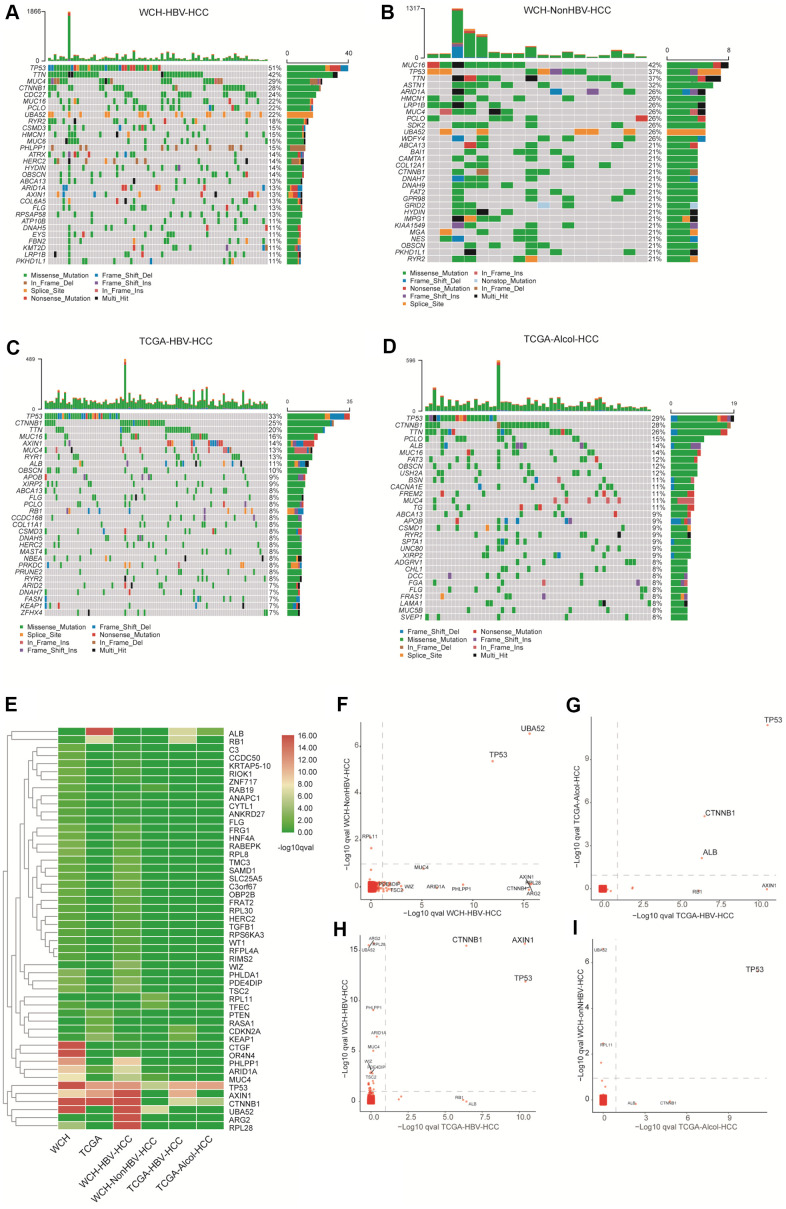
**Gene mutations in the four groups.** (**A**) The top 30 most commonly mutant genes in the WCH-HBV-HCC group. (**B**) The top 30 most commonly mutant genes in the WCH-NonHBV-HCC group. (**C**) TCGA-HBV- The top 30 most commonly mutant genes in the HCC group. (**D**) Top 30 most commonly mutant genes in the TCGA-Alcol-HCC group. (**E**) Hierarchical cluster analysis shows the q value of gene mutations in each group. The screening criteria of genes shown in Figure 2E is: q value <0.1 in at least one group. (**F**) Gene mutations in the WCH-HBV-HCC group and WCH-NonHBV-HCC group. (**G**) Gene mutations in the TCGA-Alcol-HCC group and TCGA-HBV-HCC group. (**H**) Gene mutations in the WCH-HBV-HCC group and TCGA-HBV-HCC group. (**I**) Gene mutations in the WCH-NonHBV-HCC group and TCGA-Alcol-HCC group. The q values for gene mutations are compared among different groups. Gene mutations with q values <0.1 were deemed as significant. The position of the dotted line is the cutoffs of the q value.

Similar to CNV, the main driver mutations showed significant heterogeneity in the four cohorts including WCH-HBV-HCC, WCH-NonHBV-HCC, TCGA-HBV-HCC and TCGA-Alcol-HCC groups (Figure 3 and Supplementary Figure 4). Gene with q value <0.1 was defined as significantly mutated gene. Figure 3E shows the significantly mutated genes appearing in the above groups. The genes presented in Figure 3E are those with q values <0.1 in at least one group. This study compared the significantly mutated genes in the above four groups and found that TP53 and UBA52 are the significantly mutated genes shared in the WCH-HBV-HCC group and WCH-NonHBV-HCC group (Figure 3F); TP53, CTNNB1 and ALB are significantly mutated genes shared in the TCGA-HBV-HCC group and the TCGA-Alcol-HCC group (Figure 3G); TP53, CTNNB1 and AXIN1 are the significantly mutated genes shared in the WCH-HBV-HCC group and the TCGA-HBV-HCC group (Figure 3H); TP53 is a significantly mutated gene shared in the WCH-NonHBV-HCC group and the TCGA-Alcol-HCC group (Figure 3I). Consequently, TP53 is a significantly mutated gene shared by the four groups of patients in this study, and the role of UBA52 mutations in HCC has not been studied (Figure 3F). In addition to these shared genes, a large number of unique mutations were observed in each groups (Supplementary Figure 4 and Figure 3E–3I).

### Mutation signature and mutation spectrum analysis

There are 6 types of point mutations: C>A/G>T, C>G/G>C, C>T/G>A, T>A/A>T, T>C/A>G, T>G/A>C. We analyzed the mutation spectrum of patients in the WCH-HBV-HCC group and the WCH-NonHBV-HCC group (Supplementary Figure 5), and found that the two most commonly mutation types were C>T and T>A in the WCH-HBV-HCC group, followed by T>C and C>A mutations. In contrast, in the WCH-NonHBV-HCC group, the frequencies of T>C and C>A mutations were significantly lower than the C>T and T>A mutations.

Based on results (cophenetic curves) of the Nonnegative Matrix Factorization (NMF) method (Supplementary Figures 6, 7), we found that the optimal number of clusters in both groups is 3. Finally, mutation signature analysis of 96 substitution patterns identified 3 signatures in the WCH-HBV-HCC group and WCH-NonHBV-HCC group, respectively (Figure 2A, 2B). Signature A of the WCH-HBV-HCC group and WCH-NonHBV-HCC group is a mutation signature that has been described and verified in previous studies, namely Signature 22 (characterized by dominant T>A mutations) in the COSMIC database, which is a characteristic mutation of HCC related to aristolochic acid. Signature B and Signature C in the WCH-HBV-HCC group are newly discovered mutation signatures (Figure 2A). Signature B (C>T) of the WCH-HBV-HCC group shows some similarity to the previously described Signature 1 (characterized by dominant C>T mutations) and 6 (C>T mutation; related to the loss of DNA mismatch repair function), 14 (C>T and C>A), 15 (C>T), 19 (C>T) and 23 (C>T). Signature C of WCH-HBV-HCC group has part of characteristics of COSMIC Signature 5 (T>C), 16 (T>C), 3 (shows all 6 types of mutation), 12 (T>C) and other signatures (Supplementary Figure 8A). Signature B and Signature C of the WCH-NonHBV-HCC group are also newly discovered mutation signatures (Figure 2B). Signature C (C>T) of the WCH-NonHBV-HCC group shows some mutation features of COSMIC Signature 1 (C>T) and 6 (C>T). Signature B of the WCH-NonHBV-HCC group has some similarity to COSMIC Signature 5 (T>C), 16 (T>C), 3 (all 6 mutation types appear) 4 (C>A; related to smoking) and 8 (C>A) (Supplementary Figure 8B). In addition, previous literatures reported that COSMIC Signature 24 (C>A) is a predominant signature of aflatoxin-related HCC. In this study, Signature B of WCH-HBV-HCC group and Signature C of WCH-NonHBV-HCC group have some mutation characteristics of Signature 24.

Figure 2C–2E, 2F–2H display the distribution of mutation signatures (Signature A, B and C) of all samples in the WCH-HBV-HCC group and WCH-NonHBV-HCC group, respectively. We found that samples in both groups showed obvious heterogeneity, and the dominant signature was distinct in different samples. Meanwhile, we clustered the HCC samples of the WCH-HBV-HCC and WCH-NonHBV-HCC groups according to the 30 mutation signatures identified in the COSMIC database (Supplementary Figures 9, 10). We observed that, except for Signature A (similar to COSMIC Signature 22), there were no signatures in the WCH group showed a strong similarity to the previously described signatures.

### Immune cell infiltration in different types of HCC

We analyzed the immune cell infiltration in the TCGA-HBV-HCC group and the TCGA-Alcol-HCC group based on the mRNA sequencing data (Supplementary Figure 11). Based on the specifically expressed gene set of immune cells related to innate and adaptive immune responses, using GSVA method, we calculated the relative expression of immune cells, angiogenesis and antigen-presenting machinery (APM) in the above two groups. The results showed that cases in the TCGA-HBV-HCC group and the TCGA-Alcol-HCC group had numerous T cell infiltration. Compared to cases in the TCGA-Alcol-HCC group, patients in the TCGA-HBV-HCC group had more infiltration (without statistically significance) of helper T cells (96.12% vs. 87.30%), overall T cells (68.93% vs. 57.14%) and B cells (9.71% vs. 1.59%). In contrast, the distribution of the remaining immune cells between the two groups is more similar. The above results indicate that, compared to gene alterations, the immune cell infiltration in the TCGA-HBV-HCC group and the TCGA-Alcol-HCC group is less heterogeneous. It is worth noting that a high frequency of samples in both groups have infiltration of CD8+ T cells and other types of T cells, which provides the possibility of immunotherapy.

### Association of gene mutation with immune cell infiltration in the TCGA cohorts

**Figure 4:**
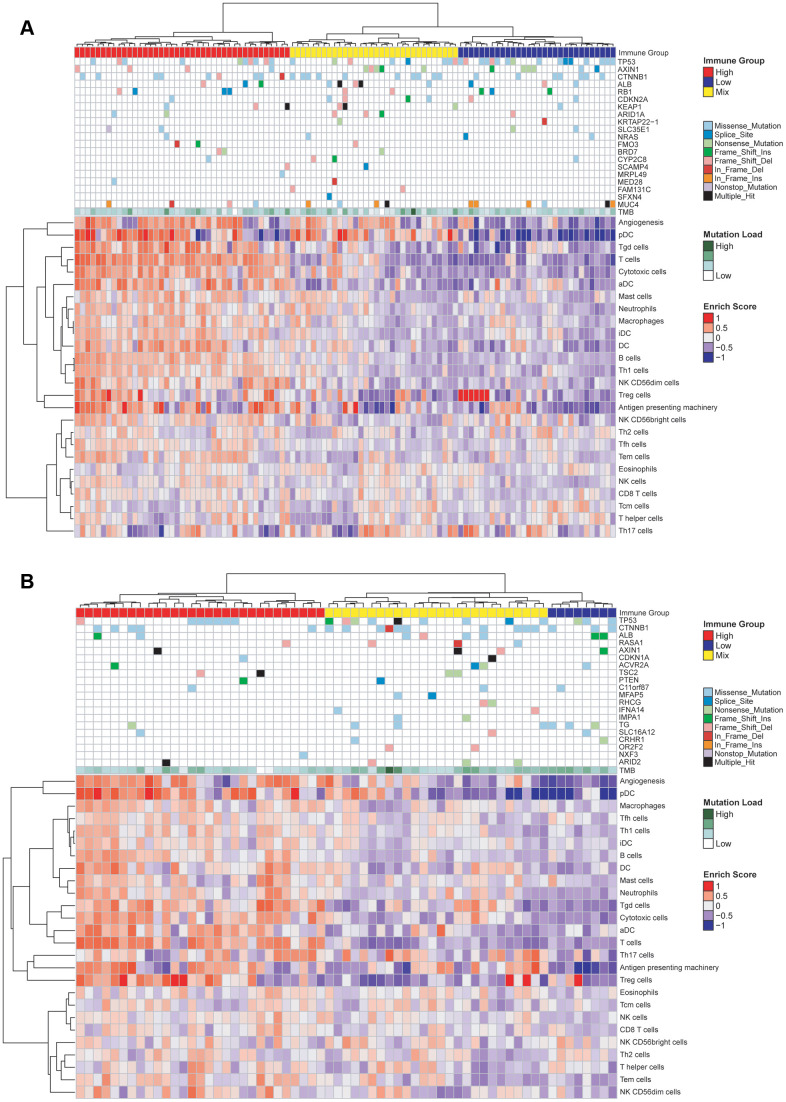
**Characterization of immune infiltration and gene mutations in the TCGA-HBV-HCC and TCGA-Alcol-HCC groups.** (**A**) Heat map shown the normalized GSVA score, tumor mutation burden (TMB) and the most common 20 mutation genes in the TCGA-HBV-HCC group. (**B**) Heat map shown the normalized GSVA score, TMB and the most common 20 mutation genes in the TCGA-Alcol-HCC group. Samples were labeled using 4 types of data: (1) Immune status (red, yellow, and blue for HIGH, MIX, and LOW); (2) Mutation burden for each sample (green); (3) The most commonly 20 mutated genes in each subtype. Mutation types including 9 types represented in different colors; (4) Enrichment score.

Using the GSVA algorithm, we calculated the distribution of 24 types of immune cells for patients in the TCGA-HBV-HCC group (Figure 4A) and TCGA-Alcol-HCC group (Figure 4B). Meanwhile, after hierarchical clustering analysis based on the GSVA score, we divided patients in the above two groups into three subgroups with distinct immune cell infiltration status (Figure 4). The immune-HIGH subgroup has the highest GSVA score and more immune cell infiltration and the GSVA score and number of tumor-infiltrating immune cell in the immune-LOW subgroup is the lowest. The GSVA score and immune cell number in the immune-MIX subgroup is between the immune-HIGH and immune-LOW groups. As shown in Figure 4, there is no significant relationship between tumor mutation burden and immune cell infiltration for patients in the TCGA-HBV-HCC and TCGA-Alcol-HCC groups. Besides, we analyzed the relationship between gene mutations and immune cell infiltration. We found that, compared to the total number of patients in the immune-HIGH and immune-MIX group, more cases in the immune-LOW subgroup had TP53 mutation (P = 0.007) (Figure 4A). In contrast, in the TCGA-Alcol-HCC group, compared to all patients in the Immune-HIGH and Immune-MIX subgroups, cases in the Immune-LOW subgroup had similar frequency of mutations in TP53, CTNNB1 and other genes (all P values> 0.05) (Figure 4B).

### Association of gene mutation with immune cell infiltration in the WCH-HBV-HCC group

**Figure 5:**
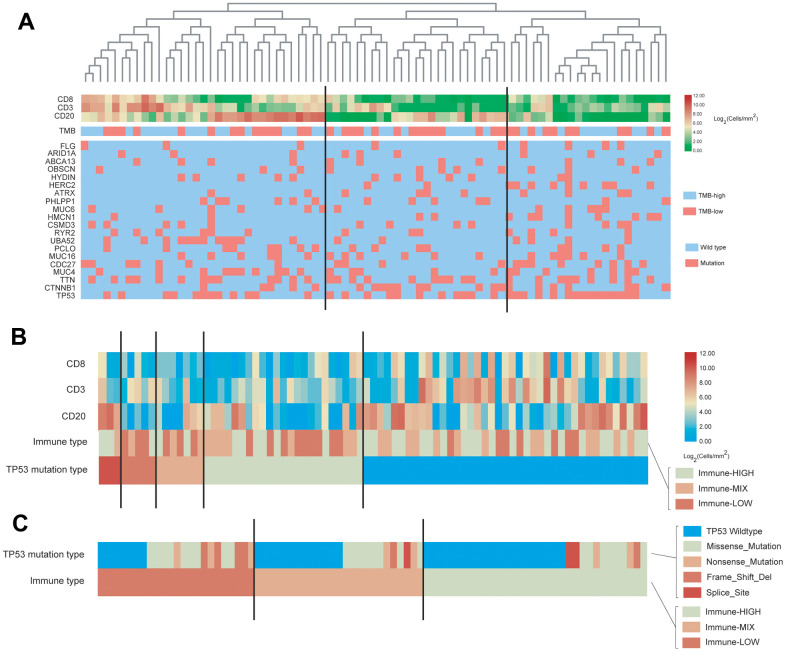
**Characterization of immune cell infiltration and gene mutations in the WCH-HBV-HCC group.** (**A**) Heat map shown the expression of immune cells by IHC staining (Log_2_ cells/mm^2^), tumor mutation burden (TMB) and the most common 20 mutation genes in the WCH-HBV-HCC group. (**B**) Heat map shown the expression of immune cells, TMB and the most common 20 mutation genes based on the TP53 mutation type in the WCH-HBV-HCC group. (**C**) Heat map shown the TP53 mutation type based on the immune type (HIGH, MIX, and LOW).

For patients in the WCH group, all tumor samples were performed immunohistochemical staining. Immune cell markers include CD3, CD8 and CD20. A representative image of the above IHC staining is shown in Supplementary Figure 12. The hematoxylin and eosin (HE) staining shows that cases in the WCH-NonHBV-HCC had distinct histopathological features (Supplementary Figure 13). Owing to the limited sample number and heterogeneous composition in the WCH-NonHBV-HCC group, in this section, we only analyzed the association of gene mutation and tumor-infiltrating immune cell in the WCH-HBV-HCC group (Figure 5). We divided the WCH-HBV-HCC group into Immune-HIGH, Immune-MIX, and Immune-LOW subgroups (Figure 5A). Similar to the TCGA cohorts, the number of most types of immune cells including CD8+ T cells, CD3+ T cells and CD20+ B cells is the highest in the immune-HIGH subgroup. Consistent to patients in the TCGA-HBV-HCC group, the tumor mutation burden in the WCH-HBV-HCC group was not significantly associated with the infiltration of immune cells in the tumor. Among patients in the WCH-HBV-HCC group, we verified the findings in the TCGA-HBV-HCC group. Finally, we found that the Immune-LOW subgroup had more TP53 mutation than cases in the immune-HIGH and immune-MIX subgroups (P = 0.049).

This study also analyzed the relationship between TP53 mutation types and immune grouping (Figure 5B). The main mutation types of TP53 in the WCH-HBV-HCC group include Missense mutation, Nonsense mutation, Frame shift deletion and Splice mutation. Figure 5A clustered the immune cells based on the above mutation types and find that, for patients in the WCH-HBV-HCC group, all frame-shift mutations, nonsense mutations, and parts of missense mutations of TP53 were associated with the decrease in the number of tumor-infiltrating immune cells.

### Association of CNV with immune cell infiltration

**Figure 6:**
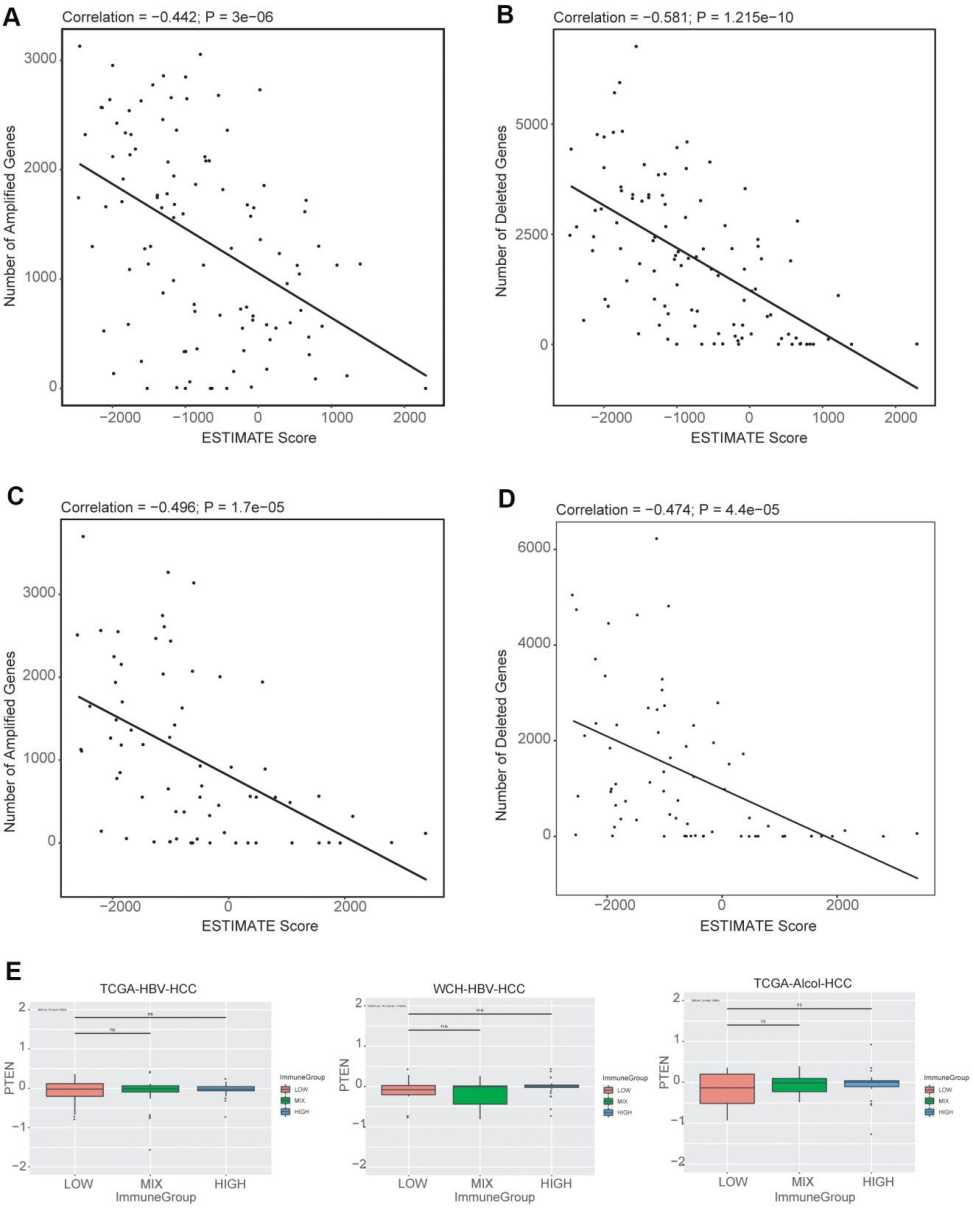
**Associations between copy number alterations and ESTIMATE immune score.** (**A**) Pearson correlation of the number of amplified genes and immune scores generated from ESTIMATE for the TCGA-HBV-HCC cohort. (**B**) Pearson correlation of the number of deleted genes and immune scores for the TCGA-HBV-HCC cohort. (**C**) Pearson correlation of the number of amplified genes and immune scores for the TCGA-Alcol-HCC cohort. (**D**) Pearson correlation of the number of deleted genes and immune scores for the TCGA-Alcol-HCC cohort; (**E**) Boxplot of PTEN copy number levels (in log_2_ level) in each of the immune infiltrate groups (HIGH, MIX and LOW) for the TCGA-HBV-HCC, WCH-HBV-HCC and TCGA-Alcol-HCC groups.

We explored the relationship between the immune infiltration score (ESTIMATE immune score) and gene copy number alterations. We observed that higher number of copy number amplifications correlated with lower ESTIMATE immune infiltrate scores in both TCGA-HBV-HCC (Figure 6A) and TCGA-Alcol-HCC (Figure 6C) groups. In addition, higher number of copy number losses were also associated with lower ESTIMATE immune scores in both TCGA-HBV-HCC (Figure 6B) and TCGA-Alcol-HCC (Figure 6D) groups (P < 0.05, Pearson’s correlation). To identify genes with CNVs that were significantly associated with immune cell infiltrate, we evaluated the relationship between the most commonly CNV-affected genes of HCC (VEGFA, FGF3/4/19, CCND1, AXIN1, CDKN2A, CDKN2B, IRF2, MAP2K3, PTEN and RB1) and ESTIMATE immune infiltrate scores. However, none of the above genes^,^ CNV were related to the immune infiltration scores. In melanoma, PTEN loss was found to be associated with the immune cell infiltration status, while on such associations were observed in HCC (TCGA-HBV-HCC, WCH-HBV-HCC and TCGA-Alcol-HCC groups) (Figure 6E).

## DISCUSSION

In this study, 100 HCC samples from the West China Hospital were tested for CNV, gene mutation, and tumor immune cell infiltration (IHC staining). We divided HCC patients in the WCH cohort into WCH-HBV-HCC group and WCH-NonHBV-HCC group. Patients in the WCH-HBV-HCC group had HBV infection, while most patients in WCH-NonHBV-HCC group had no viral hepatitis and other clear pathogenic factors. Meanwhile, this study included two types of HCC in the TCGA database (TCGA-HBV-HCC group and the TCGA-Alcol-HCC group) [1]. The WCH-HBV-HCC group and the WCH-NonHBV-HCC group represent HCC of different causes in the same population, while the WCH-HBV-HCC group and the TCGA-HBV-HCC group represent HCC in different populations with the same etiological factor. Compared with the other three groups, the TCGA-Alcol-HCC group represents HCC with different etiologies and different populations (patients in TCGA-Alcol-HCC group are mainly the White). Based on transcriptome sequencing and exon sequencing data, we analyzed the genomic and immune microenvironment profiles of the above two HCC groups in Western populations. Through the analysis and comparison of the above four types of HCC population, this study found that these four types of HCC had extensive heterogeneity at the genomic level.

First, this study analyzed the CNV status of the four groups of HCC. Previous literatures showed that CNV-Amp of chromosome 1q is the most common CNV in HCC, and it is also one of the earliest events in the development of HCC [25]. Consistent with these conclusions, we observed that CNV-Amp of chromosome 1q in all four groups. Similarly, the CNV-Amp of chromosome 8q also appeared in the above four groups. In addition to CNV-Amp, CNV-Del was also frequently found in the four groups. For example, CNV-Del of chromosome 4q exists in both WCH-HBV-HCC group and TCGA-HBV-HCC group; CNV-Del of chromosome 8 such as 8p appears in all four groups. These observations are consistent with previous studies that the deletion of chromosome 8 is closely related to the occurrence of early HCC [3, 26]. In this study, we compared the CNV levels in the TCGA-HBV-HCC group and WCH-HBV-HCC group and found that some CNVs were significantly different in the above groups. For example, focal alteration at 6p21 (gene region of VEGFA) was specifically identified in the WCH-HBV-HCC group, but not in the TCGA-HBV-HCC group. The focally amplified genes in the region of 11q13 include CCND1 (which is a common CNV-affected gene in HCC) [27], and the amplification of CCND1 was only detected in the WCH-HBV-HCC group. This study found that the CNV levels of HBV-related HCC in the Eastern and Western populations were heterogeneous. The genomic instability caused by different carcinogenic factors in HCC leads to the frequent appearance of CNV in HCC, and these CNVs may play an important role in the development and progression of HCC.

Second, this study identified high-frequency mutated genes in patients from the WCH-HBV-HCC group, WCH-NonHBV-HCC group, TCGA-HBV-HCC group, and TCGA-Alcol-HCC group. TP53 had the highest mutation frequency in HBV-related HCC of the WCH and TCGA groups. In the WCH-HBV-HCC group, more than half (51%) of patients had TP53 mutations. The mutation frequency of CTNNB1 was similar in the TCGA-HBV-HCC group and the TCGA-HBV-HCC group (28% vs. 25%). In addition, TP53 and CTNNB1 were also two genes with the highest mutation frequency in the TCGA-Alcol-HCC group, and most of the TP53 and CTNNB1 mutations appeared individually, and only a small number of patients had the above two types of mutations simultaneously [10, 12]. As mentioned above, most of patients in the WCH-NonHBV-HCC group represent a group of HCC with unknown causes. In this group, TP53 mutation and MUC16 mutation were the two most common types of gene mutation. MUC16 is a member of the mucin family glycoprotein, which has been used as a tumor marker in the early diagnosis of ovarian cancer and other tumors [28, 29]. MUC16 can promote tumorigenesis in ovarian cancer and play a role in tumor proliferation [30, 31]. Previous studies have suggested that HCC tumor cells did not produce mucin, but the latest research proved that tumor cells can produce mucins such as MUC16 in HCC with bile duct differentiation. Future researches need to further explore the role of mucin molecules such as MUC16 and MUC4 in the development of HCC [32]. In summary, TP53 is the most common genetic mutation that occurs in all four types of HCC patients. However, the most common genetic mutations in HCC, such as TP53 and CTNNB1, cannot currently be used as drug treatment targets. For solid tumors, the frequency of gene mutations that can be treated (kinase mutations, such as EGFR) is very low. The CNV-Amp of FGF19 (11q13) is a genetic change that appears in the WCH-HBV-HCC group. Currently, drugs targeting FGF19 have been developed. However, the frequency of CNV-Amp of FGF19 is still very low in the WCH-HBV-HCC group. Therefore, it is still very difficult in screening of targeted drugs that are generally effective for HCC. In the future, targeted therapy for HCC needs to be more individualized, and combinatorial approaches may achieve better therapeutic effects [33, 34].

Next, through the NMF method, this paper systematically analyzed the mutation spectrum and signatures of patients in the WCH-HBV-HCC and WCH-NonHBV-HCC groups. This study identified three types of mutation signatures in the WCH-HBV-HCC group and the WCH-NonHBV-HCC group, respectively. We found that Signature A (characterized by T>A) in the above two groups was associated with exposure to aristolochic acid (Signature 22 of the COSMIC database). The HCC patients in the WCH group mainly come from the western region of China, but HCC patients in the eastern coastal region of China have also identified the widespread existence of this mutation signature [35–37]. This is consistent with the clinically observed phenomenon [10], that is, a large proportion of patients in China have received Chinese herbal medicine containing aristolochic acid. However, previous studies only analyzed the genomic mutation profile, and there is no direct evidence that aristolochic acid causes HCC. Recently, the study published by Zeguang et al. directly proved the association of aristolochic acid and HCC [38]. The animal models in this study proved that aristolochic acid can lead to HCC in mice in a dose-dependent manner. Aristolochic acid can cause DNA damage in mice liver and form adduct with DNA. The study proves that aristolochic acid causes a typical DNA base T>A transversion in mouse liver. Clonal evolution analysis found that gene mutations caused by aristolochic acid appeared very early in the process of clonal evolution, indicating that aristolochic acid may be the key to malignant transformation of cells. In addition, we found that Signature B and C of the WCH-HBV-HCC group and Signature B and C of the WCH-NonHBV-HCC group could not correspond well with the existing Signature of the COSMIC database. The four types of Signature integrate the characteristics of some signature from the COSMIC database, which indicates that the pathogenic factors of HCC patients in the WCH group may be complex, and multiple factors have caused the occurrence of HCC. Compared to patients with HCC in eastern China [12, 35], HCC patients in the WCH group have unique mutation characteristics. The mutation signatures established in this study can classify patients in the West China Hospital effectively, compared with the existing liver cancer mutation signatures.

With the application of immunotherapy, understanding the immune microenvironment profile of HCC is crucial for screening of specific populations sensitive to immunotherapy. Through the GSVA algorithm, we compared the immune cell infiltration status in the TCGA-HBV-HCC group and the TCGA-Alcol-HCC group. We found that the number of most types of immune cells was not significantly different between the two groups, which indicated that the heterogeneity of tumor immune microenvironment may be lower than the in the genomic level, and immunotherapy holds substantial promise for tumors that are resistant to standard therapies [39]. We found that tumor mutation burden and most gene mutations have no correlation with tumor-infiltrating immune cells. This is inconsistent with the findings in some other types of solid tumors. For example, Knudsen et al. demonstrated that, in some patients with pancreatic cancer, the tumor mutation burden is positively correlated with immune cell infiltration [40]. The above-mentioned correlation was not observed in HCC, and the explanation may be that, except for several genes such as TP53 and CTNNB1, the frequency of other molecular mutation in HCC is low. In addition, due to the immunosuppressive microenvironment of HCC, tumor neoantigens produced by tumor mutations cannot cause a significant immune response within the tumor. However, we found that the total CNV levels (both amplification and deletion of genes) were significantly associated with immune cell infiltration, which is consistent with other types of tumor [41, 42]. These data highlight the need to explore multiple contributors to immune cell infiltration in HCC, and future studies are needed to illustrate the impact of gene CNV on tumor immune cell infiltration and responses to immune therapy.

This study found that the mutation of TP53 may affect the infiltration of immune cells in the tumor. In the WCH-HBV-HCC and TCGA-HBV-HCC groups, the immune-LOW subgroup had more TP53 mutations. Previous studies have found that TP53 mutations can affect the generation of anti-tumor immune responses and promote immune escape [43]. Studies have shown that TP53 mutations can predict the therapeutic effect of PD-1/PD-L1 inhibitors in non-small cell lung cancer (patients with TP53 mutation have a higher overall survival rate after immunotherapy) [44]. Prof. Wu Yilong found that, in non-small cell lung cancer, TP53 can regulate the expression of PD-L1, and the TP53 frameshift mutation predicts better immunotherapy efficacy [45]. In HCC, because there is no obvious correlation between TMB and immune infiltration, and the expression of PD-L1 in tumor is low, future research still needs to develop new markers to predict the efficacy of PD-1/PD-L1 inhibitors in HCC, and whether the TP53 mutation can be used as a predictor for the efficacy of immunotherapy in HCC needs further illustrations. In the TCGA-Alcol-HCC group, this study found that mutations such as TP53 and CTNNB1 had no significant correlation with immune cell infiltration. In the immune-LOW subgroup of the TCGA-Alcol-HCC group, the proportion of CTNNB1 mutations was higher, but there was no statistical difference. In melanoma, studies have found that the activated Wnt/β-catenin pathway affected the strength of tumor immune responses [46]. The activated Wnt pathway can down-regulate the expression of CCL4 through ATF3-dependent transcriptional inhibition, which ultimately affects the infiltration and activation of dendritic cells and CD8+ T cells [47]. In HCC (especially alcoholic HCC), it is necessary to further illustrate the relationship between CTNNB1 mutation and the immune response and the underlying mechanism behind it.

This study has some limitations. For example, the immune cell infiltration of patients in the WCH group was analyzed by immunohistochemical staining, while transcriptome sequencing data was used in the TCGA group, which may influence the accuracy of the results; In addition, the pathogenic causes of patients in the WCH-NonHBV-HCC group is unknown; The sample size of the WCH-NonHBV-HCC group is small, so we did not classify the immune infiltration status in this group. Finally, the CNV, gene mutation and mutation signatures identified in this study need to be verified by study with a larger sample size. In the future, more researches are needed to elaborate the function and significance of the molecular mutations discovered in this study in the development and progression of HCC.

## Supplementary Material

Supplementary Materials and Methods

Supplementary Figures

Supplementary Tables

## References

[1] Villanueva A. Hepatocellular carcinoma. N Engl J Med. 2019; 380:1450–62. 10.1056/NEJMra1713263 30970190

[2] Llovet JM, Zucman-Rossi J, Pikarsky E, Sangro B, Schwartz M, Sherman M, Gores G. Hepatocellular carcinoma. Nat Rev Dis Primers. 2016; 2:16018. 10.1038/nrdp.2016.18 27158749

[3] Craig AJ, von Felden J, Garcia-Lezana T, Sarcognato S, Villanueva A. Tumour evolution in hepatocellular carcinoma. Nat Rev Gastroenterol Hepatol. 2020; 17:139–52. 10.1038/s41575-019-0229-4 31792430

[4] Arzumanyan A, Reis HM, Feitelson MA. Pathogenic mechanisms in HBV- and HCV-associated hepatocellular carcinoma. Nat Rev Cancer. 2013; 13:123–35. 10.1038/nrc3449 23344543

[5] Farazi PA, DePinho RA. Hepatocellular carcinoma pathogenesis: from genes to environment. Nat Rev Cancer. 2006; 6:674–87. 10.1038/nrc1934 16929323

[6] Yang JD, Hainaut P, Gores GJ, Amadou A, Plymoth A, Roberts LR. A global view of hepatocellular carcinoma: trends, risk, prevention and management. Nat Rev Gastroenterol Hepatol. 2019; 16:589–604. 10.1038/s41575-019-0186-y 31439937 PMC6813818

[7] Forner A, Reig M, Bruix J. Hepatocellular carcinoma. Lancet. 2018; 391:1301–14. 10.1016/S0140-6736(18)30010-2 29307467

[8] Gao Q, Wang XY, Zhou J, Fan J. Multiple carcinogenesis contributes to the heterogeneity of HCC. Nat Rev Gastroenterol Hepatol. 2015; 12:13. 10.1038/nrgastro.2014.6-c1 25421581

[9] Zucman-Rossi J, Villanueva A, Nault JC, Llovet JM. Genetic landscape and biomarkers of hepatocellular carcinoma. Gastroenterology. 2015; 149:1226–39.e4. 10.1053/j.gastro.2015.05.061 26099527

[10] Ally A, Balasundaram M, Carlsen R, Chuah E, Clarke A, Dhalla N, Holt RA, Jones SJ, Lee D, Ma Y, Marra MA, Mayo M, Moore RA, et al, and Cancer Genome Atlas Research Network, electronic address: wheeler@bcm.edu. Comprehensive and integrative genomic characterization of hepatocellular carcinoma. Cell. 2017; 169:1327–41.e23. 10.1016/j.cell.2017.05.046 28622513 PMC5680778

[11] Chaisaingmongkol J, Budhu A, Dang H, Rabibhadana S, Pupacdi B, Kwon SM, Forgues M, Pomyen Y, Bhudhisawasdi V, Lertprasertsuke N, Chotirosniramit A, Pairojkul C, Auewarakul CU, et al, and TIGER-LC Consortium. Common molecular subtypes among Asian hepatocellular carcinoma and cholangiocarcinoma. Cancer Cell. 2017; 32:57–70.e3. 10.1016/j.ccell.2017.05.009 28648284 PMC5524207

[12] Gao Q, Zhu H, Dong L, Shi W, Chen R, Song Z, Huang C, Li J, Dong X, Zhou Y, Liu Q, Ma L, Wang X, et al. Integrated Proteogenomic Characterization of HBV-Related Hepatocellular Carcinoma. Cell. 2019; 179:561–77.e22. 10.1016/j.cell.2019.08.052 31585088

[13] McGlynn KA, Petrick JL, El-Serag HB. Epidemiology of Hepatocellular Carcinoma. Hepatology. 2021 (Suppl 1); 73:4–13. 10.1002/hep.31288 32319693 PMC7577946

[14] Greten TF, Lai CW, Li G, Staveley-O’Carroll KF. Targeted and immune-based therapies for hepatocellular carcinoma. Gastroenterology. 2019; 156:510–24. 10.1053/j.gastro.2018.09.051 30287171 PMC6340758

[15] Chen S, Cao Q, Wen W, Wang H. Targeted therapy for hepatocellular carcinoma: challenges and opportunities. Cancer Lett. 2019; 460:1–9. 10.1016/j.canlet.2019.114428 31207320

[16] Llovet JM, Montal R, Sia D, Finn RS. Molecular therapies and precision medicine for hepatocellular carcinoma. Nat Rev Clin Oncol. 2018; 15:599–616. 10.1038/s41571-018-0073-4 30061739 PMC12452113

[17] Zhu YJ, Zheng B, Wang HY, Chen L. New knowledge of the mechanisms of sorafenib resistance in liver cancer. Acta Pharmacol Sin. 2017; 38:614–22. 10.1038/aps.2017.5 28344323 PMC5457690

[18] Iñarrairaegui M, Melero I, Sangro B. Immunotherapy of hepatocellular carcinoma: facts and hopes. Clin Cancer Res. 2018; 24:1518–24. 10.1158/1078-0432.CCR-17-0289 29138342

[19] Dagogo-Jack I, Shaw AT. Tumour heterogeneity and resistance to cancer therapies. Nat Rev Clin Oncol. 2018; 15:81–94. 10.1038/nrclinonc.2017.166 29115304

[20] Li W, Xu L, Han J, Yuan K, Wu H. Identification and validation of tumor stromal immunotype in patients with hepatocellular carcinoma. Front Oncol. 2019; 9:664. 10.3389/fonc.2019.00664 31448222 PMC6691778

[21] Mermel CH, Schumacher SE, Hill B, Meyerson ML, Beroukhim R, Getz G. GISTIC2.0 facilitates sensitive and confident localization of the targets of focal somatic copy-number alteration in human cancers. Genome Biol. 2011; 12:R41. 10.1186/gb-2011-12-4-r41 21527027 PMC3218867

[22] Talevich E, Shain AH, Botton T, Bastian BC. CNVkit: genome-wide copy number detection and visualization from targeted DNA sequencing. PLoS Comput Biol. 2016; 12:e1004873. 10.1371/journal.pcbi.1004873 27100738 PMC4839673

[23] Lawrence MS, Stojanov P, Polak P, Kryukov GV, Cibulskis K, Sivachenko A, Carter SL, Stewart C, Mermel CH, Roberts SA, Kiezun A, Hammerman PS, McKenna A, et al. Mutational heterogeneity in cancer and the search for new cancer-associated genes. Nature. 2013; 499:214–18. 10.1038/nature12213 23770567 PMC3919509

[24] Hama N, Totoki Y, Miura F, Tatsuno K, Saito-Adachi M, Nakamura H, Arai Y, Hosoda F, Urushidate T, Ohashi S, Mukai W, Hiraoka N, Aburatani H, et al. Epigenetic landscape influences the liver cancer genome architecture. Nat Commun. 2018; 9:1643. 10.1038/s41467-018-03999-y 29691395 PMC5915380

[25] Pelosi E, Castelli G, Testa U. Pancreatic Cancer: Molecular Characterization, Clonal Evolution and Cancer Stem Cells. Biomedicines. 2017; 5:65. 10.3390/biomedicines5040065 29156578 PMC5744089

[26] Wang K, Lim HY, Shi S, Lee J, Deng S, Xie T, Zhu Z, Wang Y, Pocalyko D, Yang WJ, Rejto PA, Mao M, Park CK, Xu J. Genomic landscape of copy number aberrations enables the identification of oncogenic drivers in hepatocellular carcinoma. Hepatology. 2013; 58:706–17. 10.1002/hep.26402 23505090

[27] Sawey ET, Chanrion M, Cai C, Wu G, Zhang J, Zender L, Zhao A, Busuttil RW, Yee H, Stein L, French DM, Finn RS, Lowe SW, Powers S. Identification of a therapeutic strategy targeting amplified FGF19 in liver cancer by Oncogenomic screening. Cancer Cell. 2011; 19:347–58. 10.1016/j.ccr.2011.01.040 21397858 PMC3061399

[28] Timmermans M, Zwakman N, Sonke GS, Van de Vijver KK, Duk MJ, van der Aa MA, Kruitwagen RF. Perioperative change in CA125 is an independent prognostic factor for improved clinical outcome in advanced ovarian cancer. Eur J Obstet Gynecol Reprod Biol. 2019; 240:364–69. 10.1016/j.ejogrb.2019.07.010 31400565

[29] Sha R, Badhulika S. Recent advancements in fabrication of nanomaterial based biosensors for diagnosis of ovarian cancer: a comprehensive review. Mikrochim Acta. 2020; 187:181. 10.1007/s00604-020-4152-8 32076837

[30] Reinartz S, Failer S, Schuell T, Wagner U. CA125 (MUC16) gene silencing suppresses growth properties of ovarian and breast cancer cells. Eur J Cancer. 2012; 48:1558–69. 10.1016/j.ejca.2011.07.004 21852110

[31] Liu Q, Cheng Z, Luo L, Yang Y, Zhang Z, Ma H, Chen T, Huang X, Lin SY, Jin M, Li Q, Li X. C-terminus of MUC16 activates Wnt signaling pathway through its interaction with β-catenin to promote tumorigenesis and metastasis. Oncotarget. 2016; 7:36800–13. 10.18632/oncotarget.9191 27167110 PMC5095040

[32] Kasprzak A, Adamek A. Mucins: the old, the new and the promising factors in hepatobiliary carcinogenesis. Int J Mol Sci. 2019; 20:1288. 10.3390/ijms20061288 30875782 PMC6471604

[33] Nault JC, Galle PR, Marquardt JU. The role of molecular enrichment on future therapies in hepatocellular carcinoma. J Hepatol. 2018; 69:237–47. 10.1016/j.jhep.2018.02.016 29505843

[34] Llovet JM, Villanueva A, Lachenmayer A, Finn RS. Advances in targeted therapies for hepatocellular carcinoma in the genomic era. Nat Rev Clin Oncol. 2015; 12:408–24. 10.1038/nrclinonc.2015.103 26054909

[35] Zhou SL, Zhou ZJ, Hu ZQ, Song CL, Luo YJ, Luo CB, Xin HY, Yang XR, Shi YH, Wang Z, Huang XW, Cao Y, Fan J, Zhou J. Genomic sequencing identifies WNK2 as a driver in hepatocellular carcinoma and a risk factor for early recurrence. J Hepatol. 2019; 71:1152–63. 10.1016/j.jhep.2019.07.014 31349001

[36] Huang A, Zhao X, Yang XR, Li FQ, Zhou XL, Wu K, Zhang X, Sun QM, Cao Y, Zhu HM, Wang XD, Yang HM, Wang J, et al. Circumventing intratumoral heterogeneity to identify potential therapeutic targets in hepatocellular carcinoma. J Hepatol. 2017; 67:293–301. 10.1016/j.jhep.2017.03.005 28323123

[37] Dong LQ, Peng LH, Ma LJ, Liu DB, Zhang S, Luo SZ, Rao JH, Zhu HW, Yang SX, Xi SJ, Chen M, Xie FF, Li FQ, et al. Heterogeneous immunogenomic features and distinct escape mechanisms in multifocal hepatocellular carcinoma. J Hepatol. 2020; 72:896–908. 10.1016/j.jhep.2019.12.014 31887370

[38] Lu ZN, Luo Q, Zhao LN, Shi Y, Wang N, Wang L, Han ZG. The mutational features of aristolochic acid-induced mouse and human liver cancers. Hepatology. 2020; 71:929–42. 10.1002/hep.30863 31692012

[39] Tahmasebi Birgani M, Carloni V. Tumor Microenvironment, a Paradigm in Hepatocellular Carcinoma Progression and Therapy. Int J Mol Sci. 2017; 18:405. 10.3390/ijms18020405 28216578 PMC5343939

[40] Knudsen ES, Vail P, Balaji U, Ngo H, Botros IW, Makarov V, Riaz N, Balachandran V, Leach S, Thompson DM, Chan TA, Witkiewicz AK. Stratification of pancreatic ductal adenocarcinoma: combinatorial genetic, stromal, and immunologic markers. Clin Cancer Res. 2017; 23:4429–40. 10.1158/1078-0432.CCR-17-0162 28348045 PMC5951386

[41] Roh W, Chen PL, Reuben A, Spencer CN, Prieto PA, Miller JP, Gopalakrishnan V, Wang F, Cooper ZA, Reddy SM, Gumbs C, Little L, Chang Q, et al. Integrated molecular analysis of tumor biopsies on sequential CTLA-4 and PD-1 blockade reveals markers of response and resistance. Sci Transl Med. 2017; 9:eaah3560. 10.1126/scitranslmed.aah3560 28251903 PMC5819607

[42] Wu CC, Beird HC, Andrew Livingston J, Advani S, Mitra A, Cao S, Reuben A, Ingram D, Wang WL, Ju Z, Hong Leung C, Lin H, Zheng Y, et al. Immuno-genomic landscape of osteosarcoma. Nat Commun. 2020; 11:1008. 10.1038/s41467-020-14646-w 32081846 PMC7035358

[43] Li Y, Zhang MC, Xu XK, Zhao Y, Mahanand C, Zhu T, Deng H, Nevo E, Du JZ, Chen XQ. Functional diversity of p53 in human and wild animals. Front Endocrinol (Lausanne). 2019; 10:152. 10.3389/fendo.2019.00152 30915036 PMC6422910

[44] Assoun S, Theou-Anton N, Nguenang M, Cazes A, Danel C, Abbar B, Pluvy J, Gounant V, Khalil A, Namour C, Brosseau S, Zalcman G. Association of TP53 mutations with response and longer survival under immune checkpoint inhibitors in advanced non-small-cell lung cancer. Lung Cancer. 2019; 132:65–71. 10.1016/j.lungcan.2019.04.005 31097096

[45] Dong ZY, Zhong WZ, Zhang XC, Su J, Xie Z, Liu SY, Tu HY, Chen HJ, Sun YL, Zhou Q, Yang JJ, Yang XN, Lin JX, et al. Potential predictive value of TP53 and KRAS mutation status for response to PD-1 blockade immunotherapy in lung adenocarcinoma. Clin Cancer Res. 2017; 23:3012–24. 10.1158/1078-0432.CCR-16-2554 28039262

[46] Pai SG, Carneiro BA, Mota JM, Costa R, Leite CA, Barroso-Sousa R, Kaplan JB, Chae YK, Giles FJ. Wnt/beta-catenin pathway: modulating anticancer immune response. J Hematol Oncol. 2017; 10:101. 10.1186/s13045-017-0471-6 28476164 PMC5420131

[47] Zhang XC, Wang J, Shao GG, Wang Q, Qu X, Wang B, Moy C, Fan Y, Albertyn Z, Huang X, Zhang J, Qiu Y, Platero S, et al. Comprehensive genomic and immunological characterization of Chinese non-small cell lung cancer patients. Nat Commun. 2019; 10:1772. 10.1038/s41467-019-09762-1 30992440 PMC6467893

