# Supplementary material for: Comprehensive analysis of genomic and immunological profiles in Chinese and Western hepatocellular carcinoma populations
Source: Aging (Albany NY). 2021 Apr 18;13(8):11564–94. doi: 10.18632/aging.202853 (PMC8109067; doi:10.18632/aging.202853)
Supplement: Supplementary Materials and Methods [file aging-13-202853-s001.pdf]

SUPPLEMENTARY MATERIALS AND METHODS

Analysis of tumor infiltrating immune cells in the TCGA-HBV-HCC and TCGA-Alcol-HCC cohorts

The gene sets representing different immune cells were obtained from previous literatures [1, 2]. A total of 26 gene sets were identified, and 11 of which represent innate immune cells: dendritic cells (DC), activated dendritic cells (aDC), plasma cell-like dendritic cells (pDC), immature trees Identical cells (iDC), mast cells, neutrophils, macrophages, eosinophils, NK cells, CD56bright NK cells and CD56dim NK cells; 13 types of adaptive immune cells: T cells, cytotoxic cells, B cells, and helper T cells 17 (Th17 cells), Regulatory T cells (Treg cells), helper T cells, helper T cells type 1 (Th1 cells), helper T cells type 2 (Th2 cells), follicular helper T cells (Tfh cells), γδ T cells (Tgd cells), CD8 T cells, effect memory T cells (Tem cells) and central memory T cells (Tcm cells); The remaining two represent angiogenesis and antigen presentation System. Cytotoxic cells include parts of CD8 + T cells, some γδ T cells and some NK cells. Tumor RNA-seq data of 69 cases of alcoholic HCC and 106 cases of HBV-related HCC in the TCGA database were used to analyze tumor-infiltrating immune cell. In this study, the Gene Set Variation Analysis (GSVA) algorithm was used to calculate the immune score (GSVA package). The RNA sequencing data of all gene sets was first standardized by Z-score, then all patients were sorted according to Z-score, and finally the expression of each immune cell is calculated by the GSVA algorithm. False discovery rate (FDR; q value) <10% is considered to be significantly enriched for this type of immune cell. In the heat map, we classified all samples into immune-High, immune-Mix, and immune-Low groups based on the number of immune cell infiltration, especially T cells, B cells and cytotoxic cells.

REFERENCES

1. Zhang XC, Wang J, Shao GG, Wang Q, Qu X, Wang B, Moy C, Fan Y, Albertyn Z, Huang X, Zhang J, Qiu Y, Platero S, et al. Comprehensive genomic and immunological characterization of Chinese non-small cell lung cancer patients. Nat Commun. 2019; 10:1772. https://doi.org/10.1038/s41467-019-09762-1 PMID:30992440

2. Bindea G, Mlecnik B, Tosolini M, Kirilovsky A, Waldner M, Obenauf AC, Angell H, Fredriksen T, Lafontaine L, Berger A, Bruneval P, Fridman WH, Becker C, et al. Spatiotemporal dynamics of intratumoral immune cells reveal the immune landscape in human cancer. Immunity. 2013; 39:782–95. https://doi.org/10.1016/j.immuni.2013.10.003 PMID:24138885
