# Supplementary material for: Comprehensive analysis of genomic and immunological profiles in Chinese and Western hepatocellular carcinoma populations
Source: Aging (Albany NY). 2021 Apr 18;13(8):11564–94. doi: 10.18632/aging.202853 (PMC8109067; doi:10.18632/aging.202853)
Supplement: Supplementary Figures [file aging-13-202853-s002.pdf]

SUPPLEMENTARY FIGURES

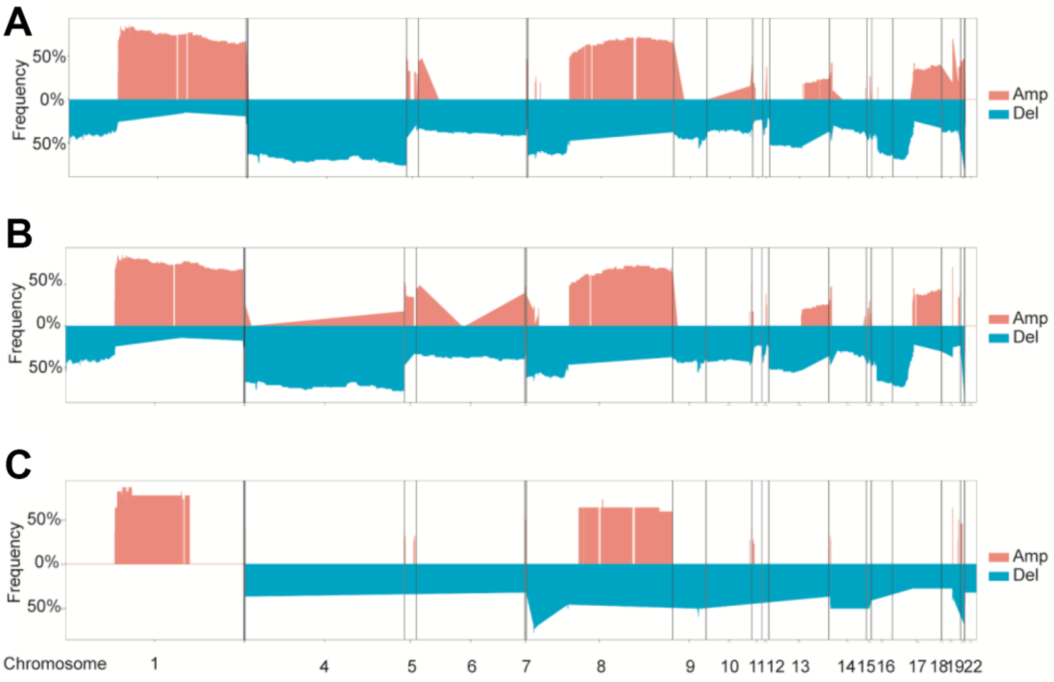

Supplementary Figure 1. Overview of copy number variation in the WCH group (**A**) All patients in the WCH group; (**B**) Patients with hepatitis B (WCH-HBV-HCC group); (**C**) Patients without hepatitis B (WCH-NonHBV-HCC group). The red color represents copy number amplification, and the green represents copy number deletion.

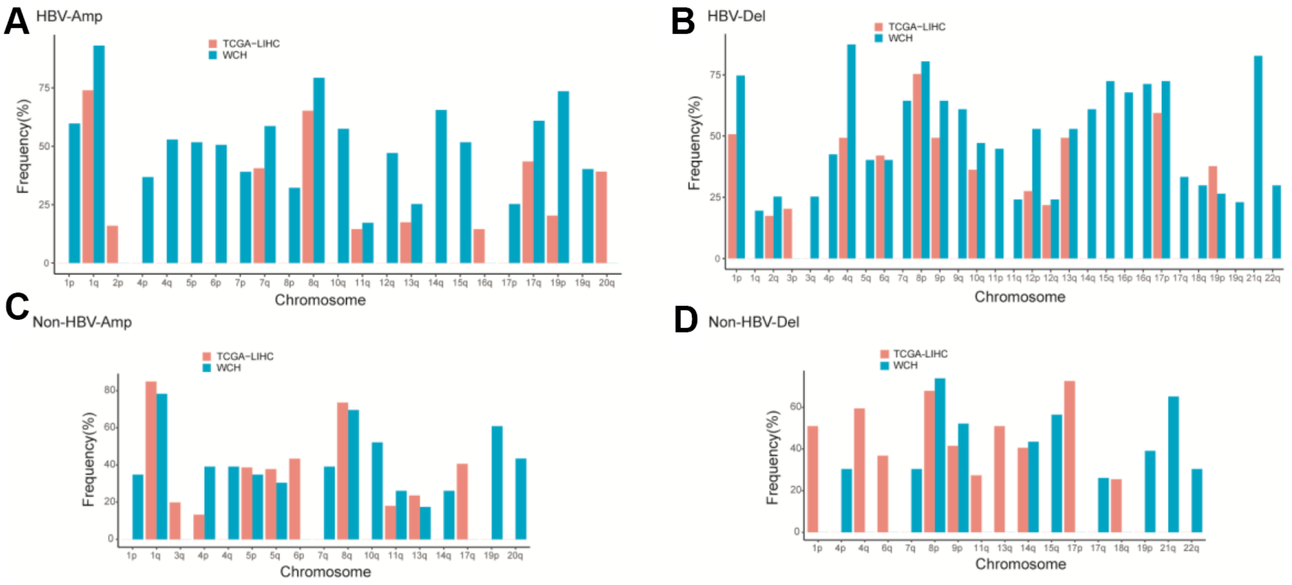

Supplementary Figure 2. Comparison of arm level copy number alterations between WCH and TCGA cohorts. (**A**) Amplification frequencies of WCH-HBV-HCC versus TCGA-HBV-HCC group; (**B**) Deletion frequencies of WCH-HBV-HCC versus TCGA-HBV-HCC group; (**C**) Amplification frequencies of WCH-NonHBV-HCC versus TCGA-Alcol-HCC group; (**D**) Deletion frequencies of TCGA-Alcol-HCC versus WCH-NonHBV-HCC group.

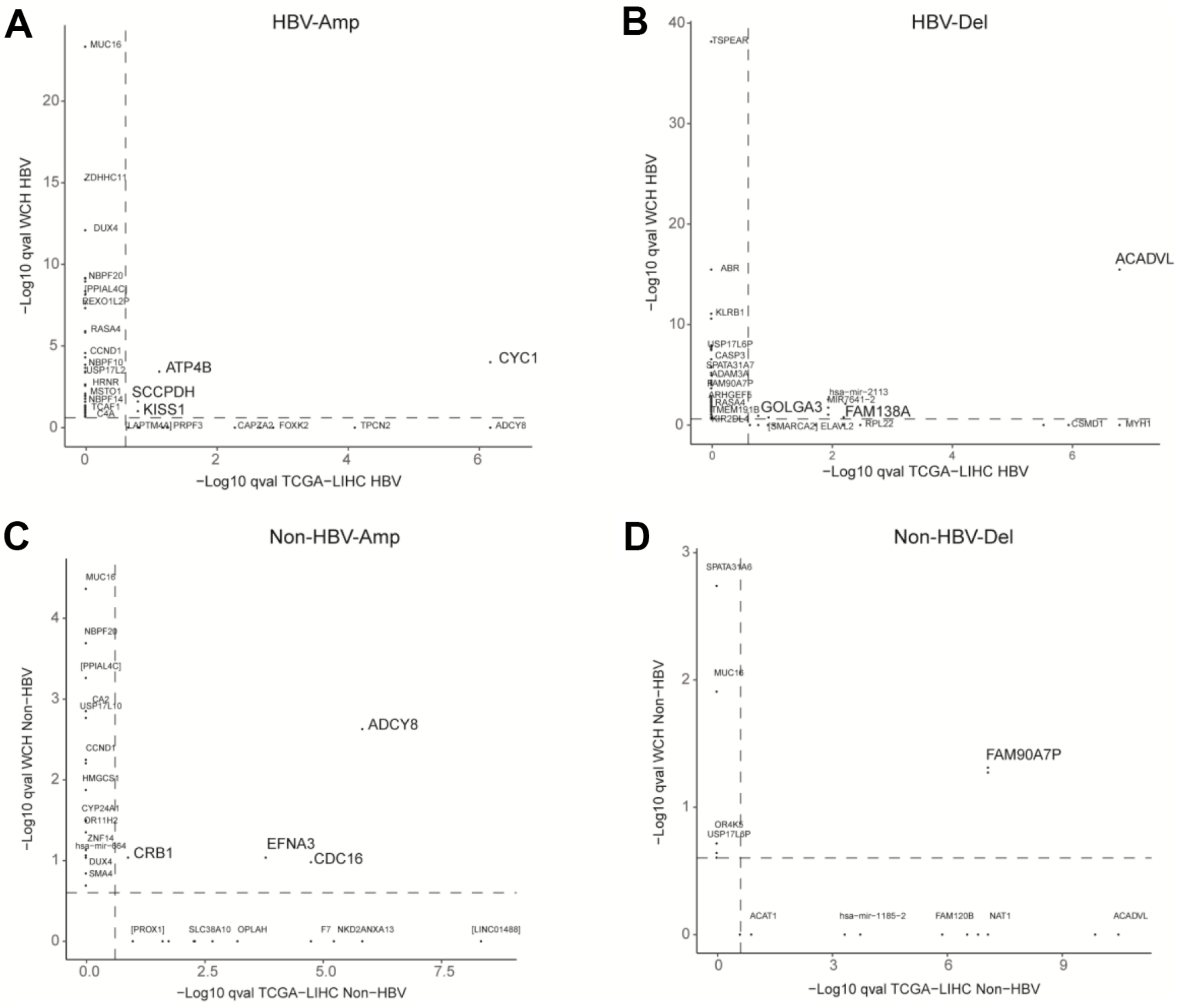

Supplementary Figure 3. The focal CNV profile between the WCH and TCGA were compared to identify novel focal events. (**A**) Focal amplifications in the WCH-HBV-HCC and TCGA-HBV-HCC groups. (**B**) Focal deletions in the WCH-HBV-HCC and TCGA-HBV-HCC groups. (**C**) Focal amplifications in the WCH-NonHBV-HCC and TCGA-NonHBV-HCC groups. (**D**) Focal deletions in the WCH-NonHBV-HCC and TCGA-NonHBV-HCC groups. The q values for amplifications (**A**, **C**) and deletions (**B**, **D**) in the WCH group were plotted against q values from the TCGA cohort. CNVs with q values <0.25 were deemed as significant. Owing to the similar q values of a large number of genes, we only showed parts of representative genes in this figure. All shared and unique genes among the above groups are shown in the Supplementary data file 2-5. The dashed line are q value cutoffs.

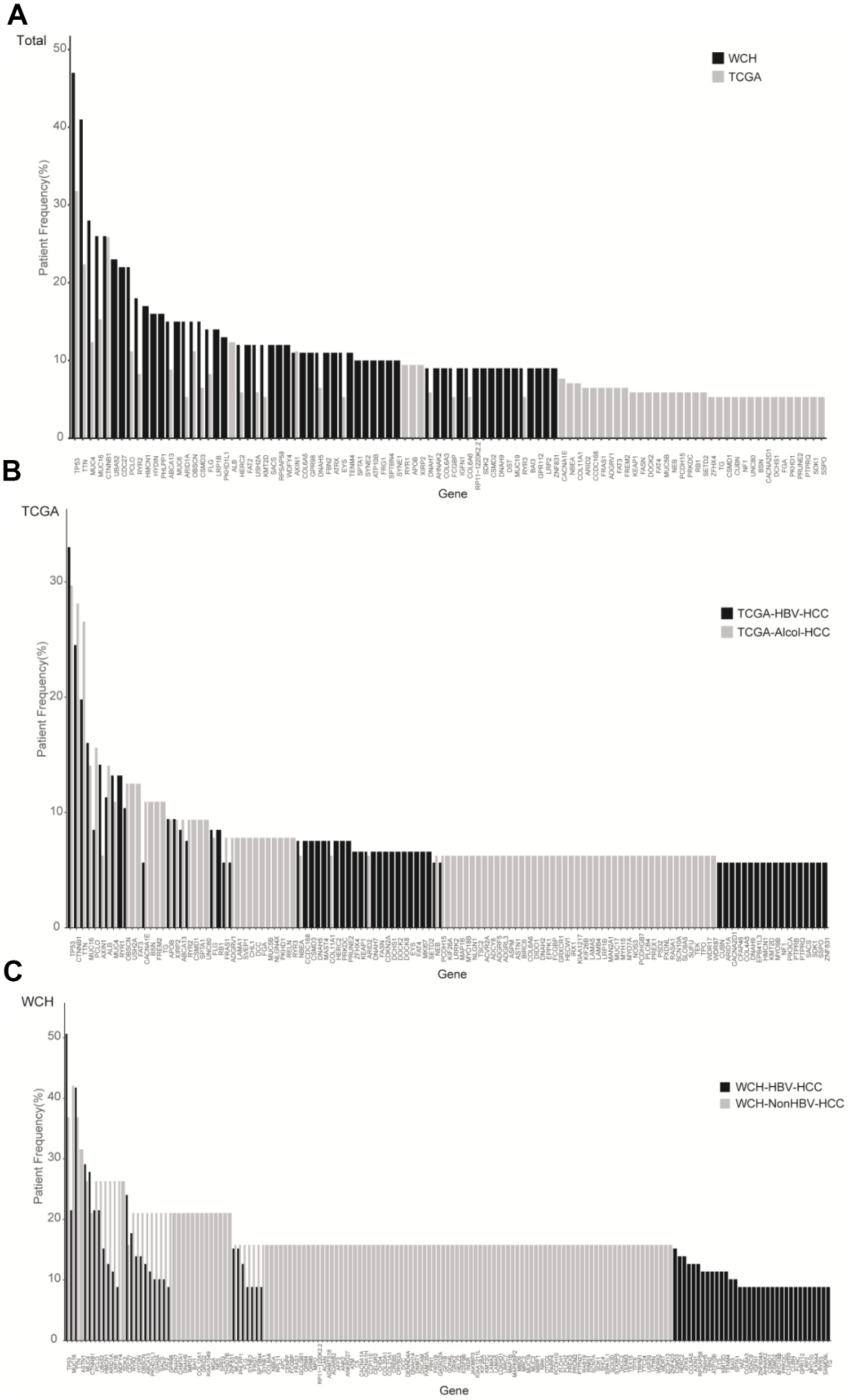

Supplementary Figure 4. Comparison of the mutation frequencies of significantly mutated gene between the WCH and TCGA cohorts. (**A**) The total WCH and TCGA cohorts; (**B**) TCGA-HBV-HCC and TCGA-Alcol-HCC groups; (**C**) WCH-HBV-HCC and WCH-NonHBV-HCC groups.

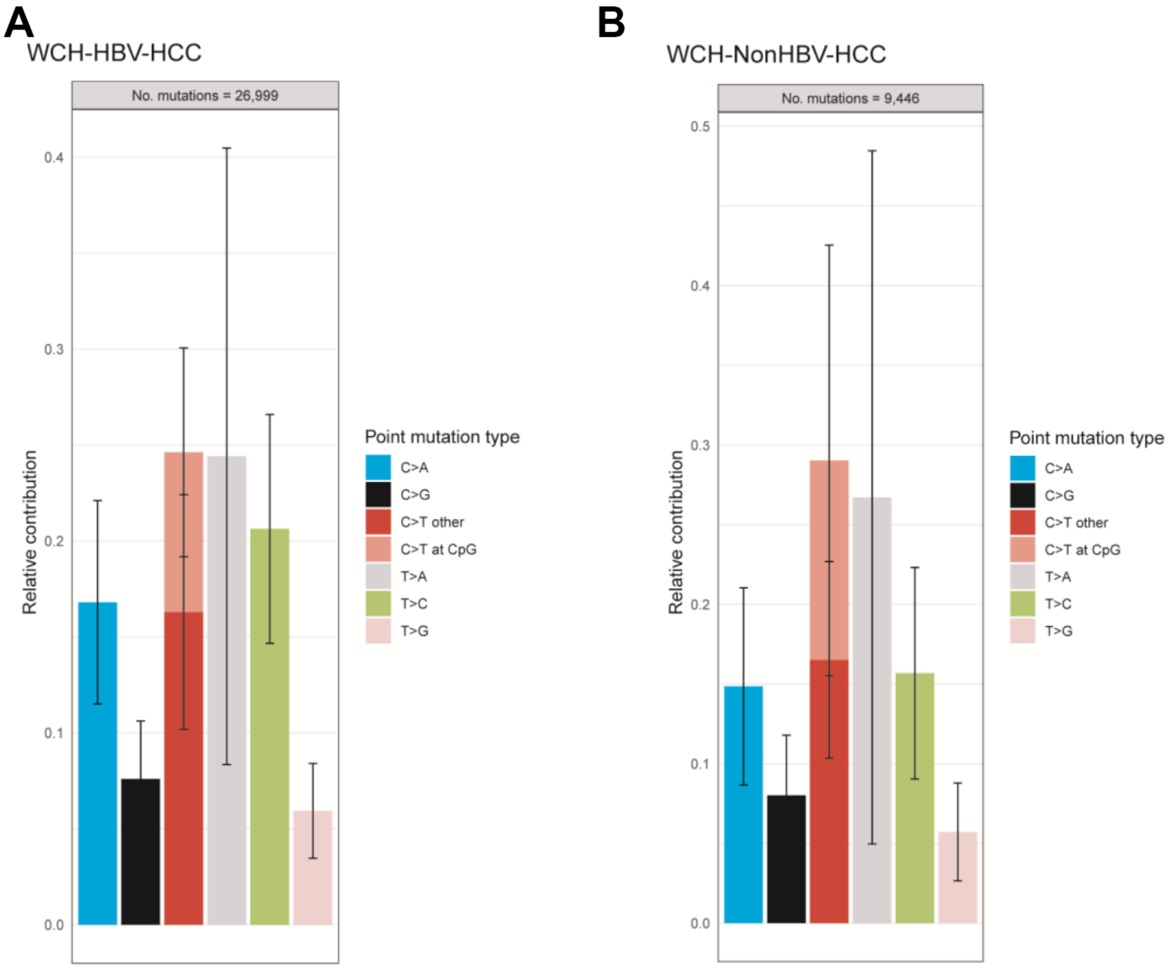

Supplementary Figure 5. Mutation spectrum of patients in WCH group. (**A**) WCH-HBV-HCC group; (**B**) WCH-NonHBV-HCC group.

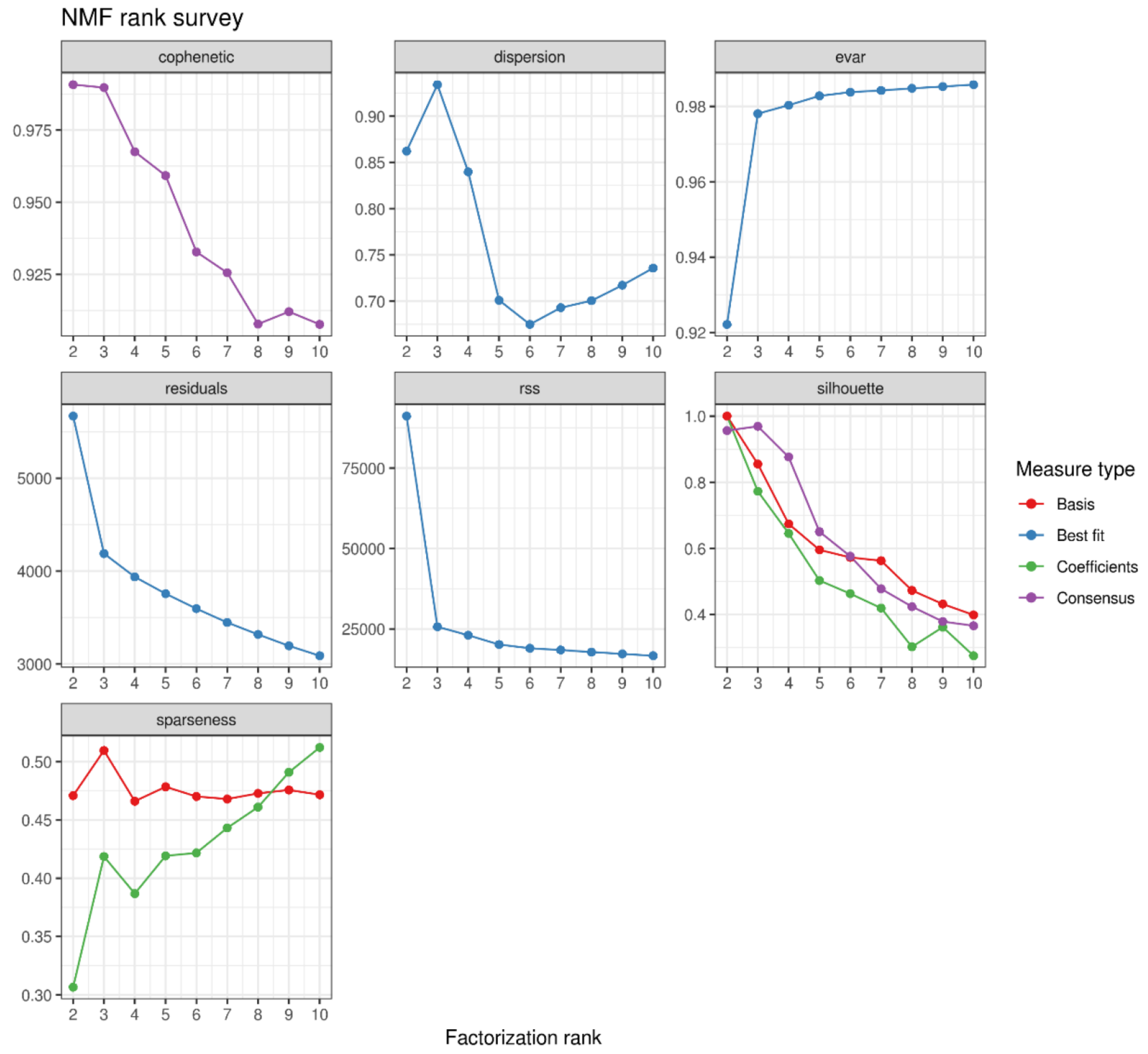

Supplementary Figure 6. Calculating the optimal clustering value based on the NMF algorithm in the WCH-HBV-HCC group. Cophenetic refers to correlation coefficient; Dispersion is the dispersion coefficient (evaluation of the repeatability of the NMF results); Evar is used to evaluation of the interpretation effect of the NMF model to the matrix differences; Silhouette is aimed to evaluate the stability of the model; Sparseness is used to calculate the sparsity of the matrix. RSS, Residual Sum of Squares.

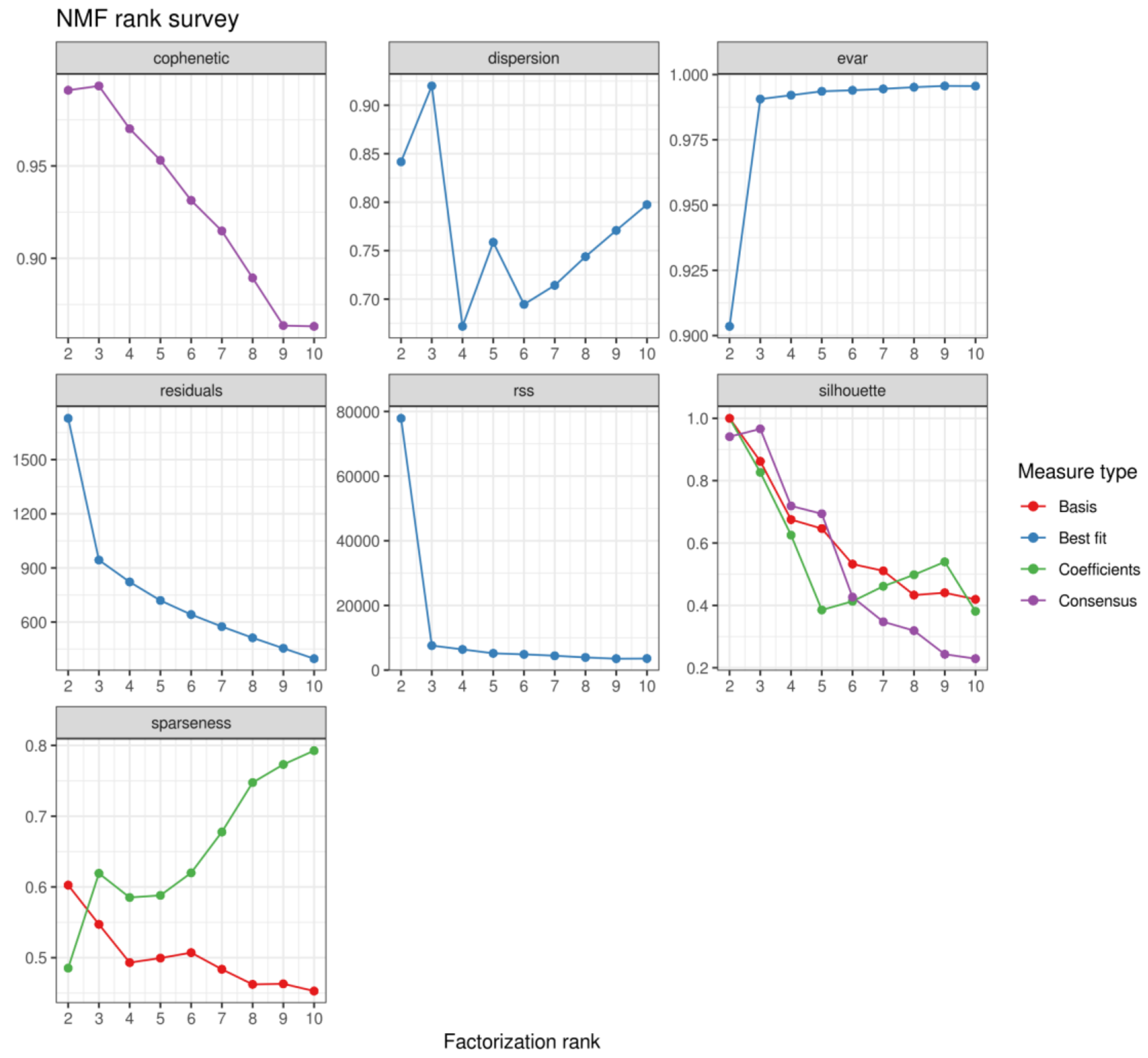

Supplementary Figure 7. Calculating the optimal clustering value based on the NMF algorithm in the WCH-NonHBV-HCC group. Cophenetic refers to correlation coefficient; Dispersion is the dispersion coefficient (evaluation of the repeatability of the NMF results); Evar is used to evaluation of the interpretation effect of the NMF model to the matrix differences; Silhouette is aimed to evaluate the stability of the model; Sparseness is used to calculate the sparsity of the matrix. RSS, Residual Sum of Squares.

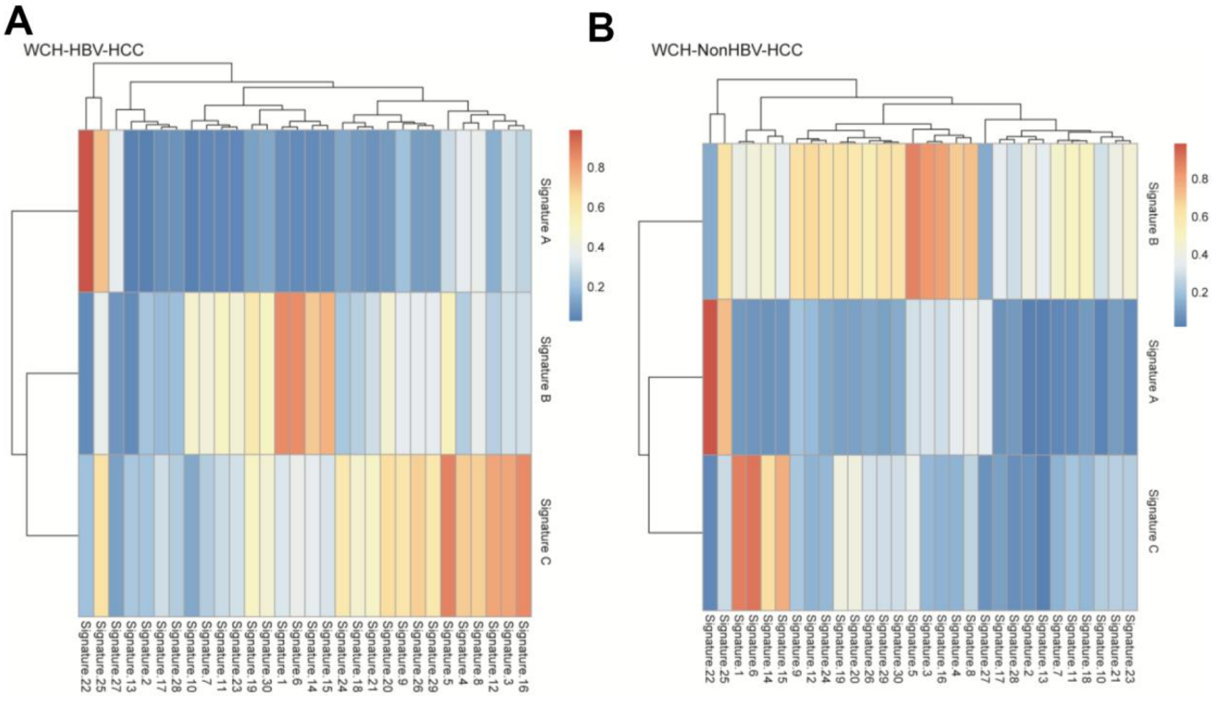

Supplementary Figure 8. Association of the mutation Signatures identified in this study with the existing mutation signatures of the COSMIC database. (**A**) WCH-HBV-HCC group; (**B**) WCH-NonHBV-HCC group.

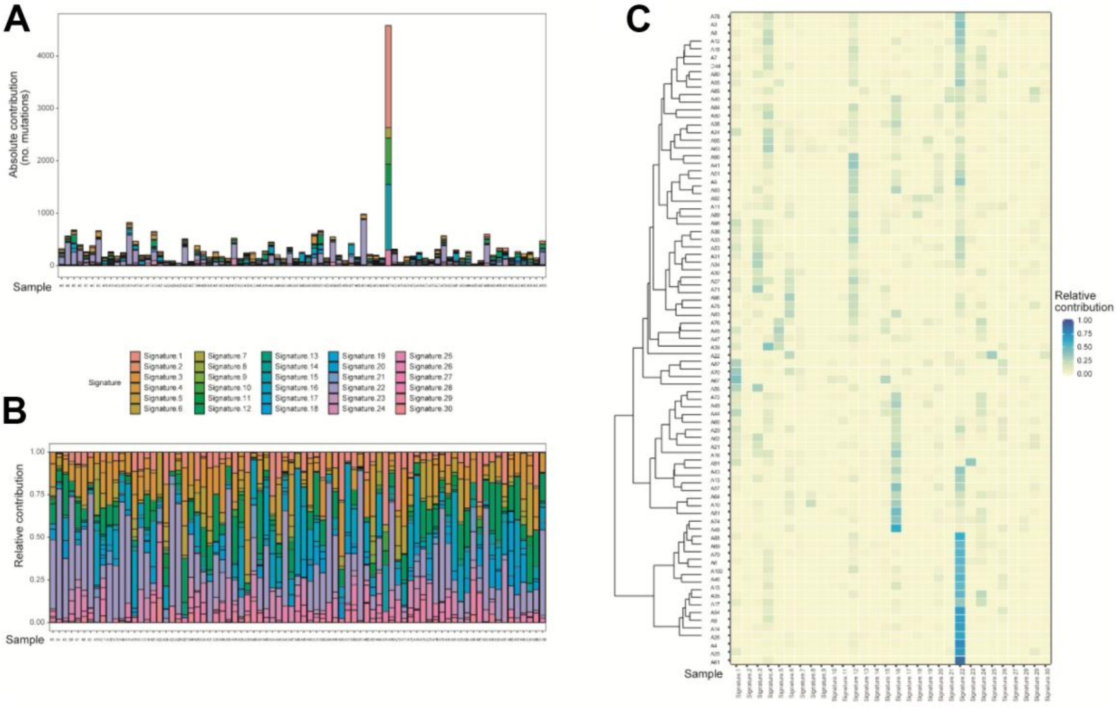

Supplementary Figure 9. Distribution of the 30 mutation Signatures of the COSMIC database among all samples in the WCH-HBV-HCC group (**A**) The contributions of the 30 mutational signatures to tumors in the WCH-HBV-HCC group. The sample names are displayed on the horizontal axis, whereas the vertical axis depicts the number of mutations of samples in the WCH-HBV-HCC group; (**B**) The relative contribution of the 30 Signatures in samples from the WCH-HBV-HCC group; (**C**) The distribution of the 30 mutation Signatures in the WCH-HBV-HCC group.

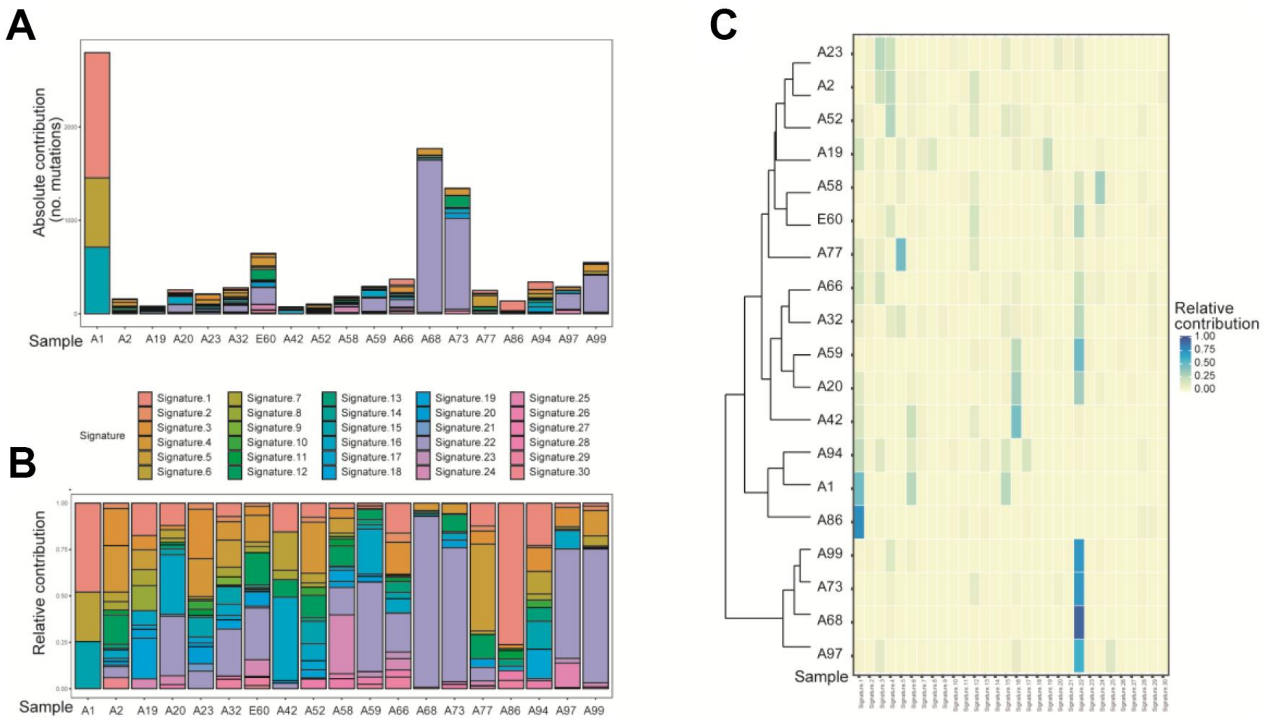

Supplementary Figure 10. Distribution of the 30 mutation Signatures of the COSMIC database among all samples in the WCH-NonHBV-HCC group (**A**) The contributions of the 30 mutational signatures to tumors in the WCH-NonHBV-HCC group. The sample names are displayed on the horizontal axis, whereas the vertical axis depicts the number of mutations of samples in the WCH-NonHBV-HCC group; (**B**) The relative contribution of the 30 Signatures in samples from the WCH-NonHBV-HCC group; (**C**) The distribution of the 30 mutation Signatures in the WCH-NonHBV-HCC group.

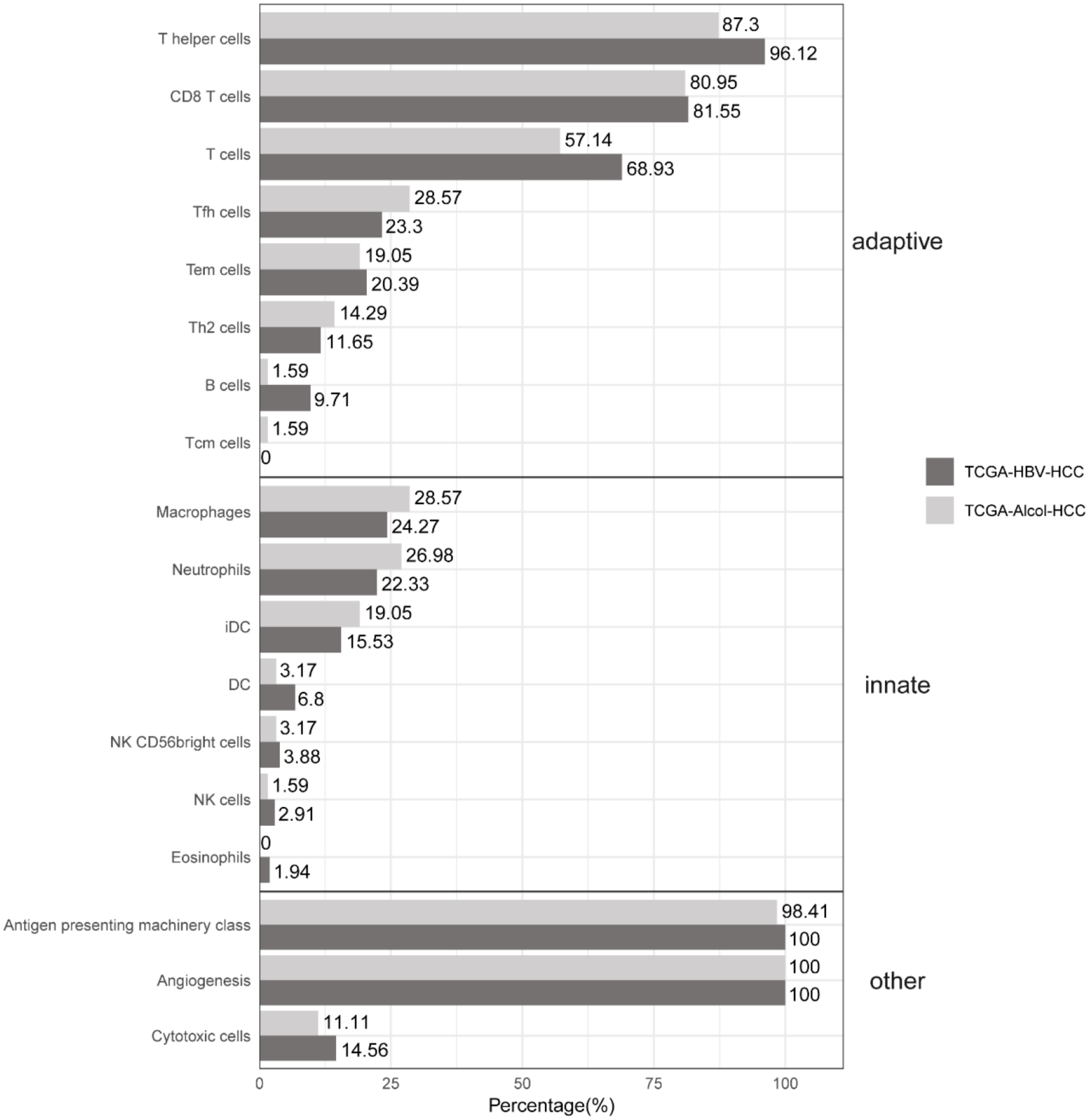

Supplementary Figure 11. Comparison of Tumor-infiltrating lymphocytes enrichment profile in the TCGA-HBV-HCC group and TCGA-Alcol-HCC group. Percentage of cases with enriched immune cell signatures were calculated using the GSVA and pre-rank Gene Set Enrichment Analysis (GSEA) methods (see Supplementary Methods). The GSEA was utilized to calculate enrichment score, while the pre-rank GSEA was used to calculate FDR values and for each immune cell signature, enrichment is defined as q-value ≤0.1. The black bars indicate the percentage of patients having significant enrichment for the given immune cell type in the TCGA-HBV-HCC group, while gray bars represent the percentage in the TCGA-Alcol-HCC group. Immune cell signatures were classified into adaptive, innate and other. Source data are provided in the Supplementary data file 16.

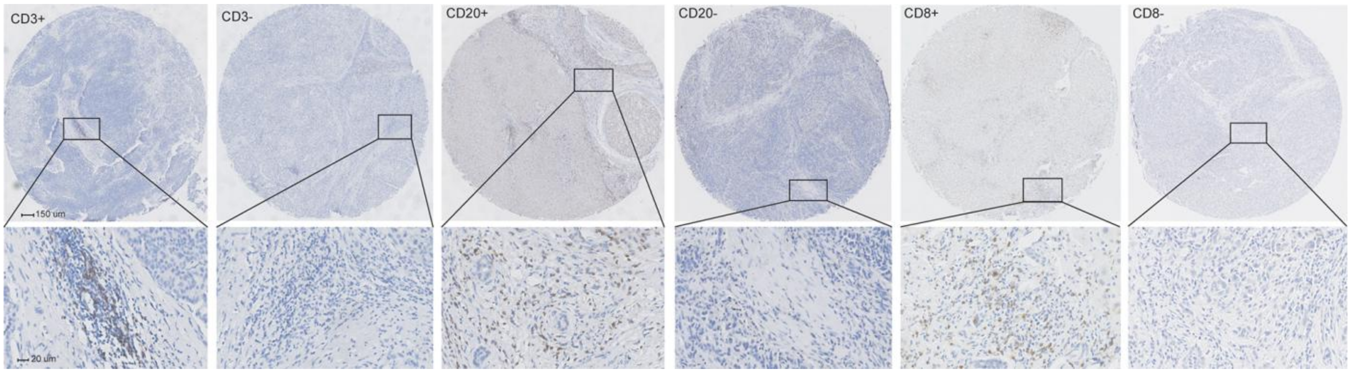

Supplementary Figure 12. Representative images of immunohistochemical staining of HCC samples in the WCH group. These immune markers are CD3+ T cells, CD20+ T cells and CD8+ B cells.

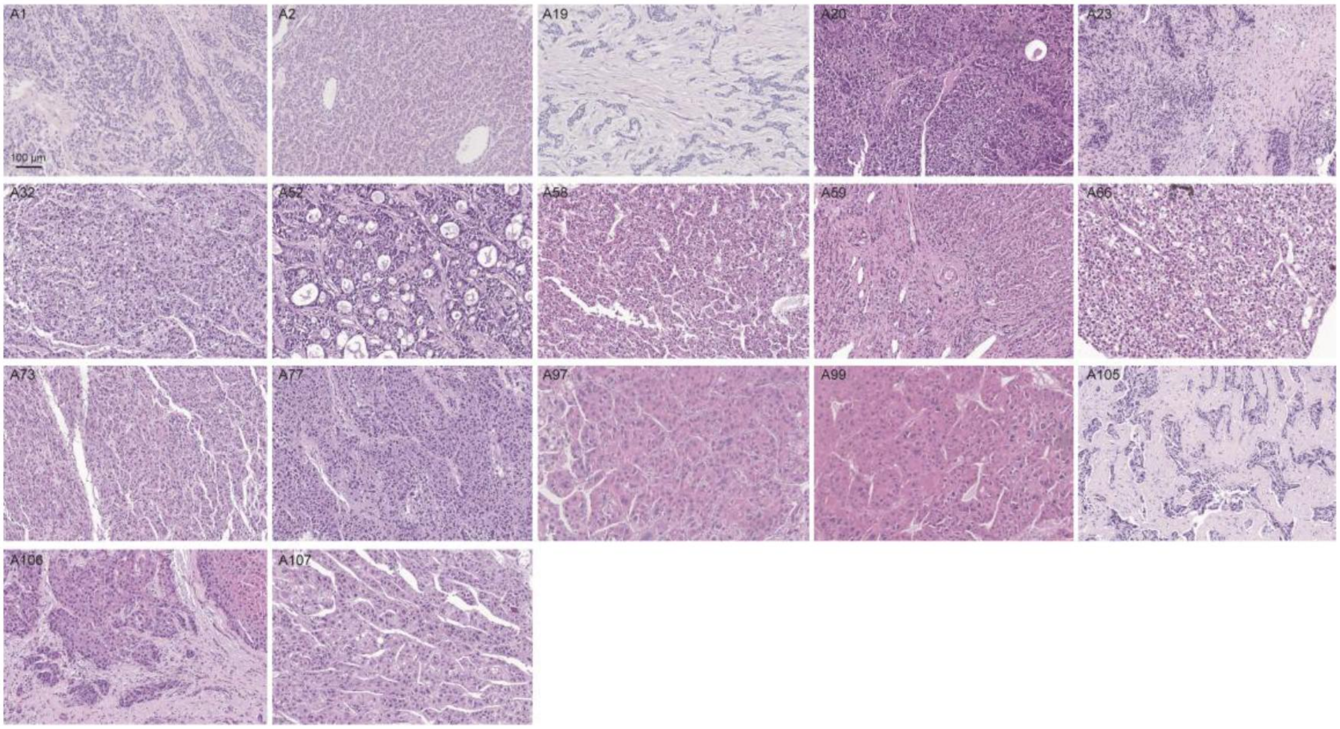

Supplementary Figure 13. Representative images of hematein-eosin staining of HCC samples in the WCH-NonHBV-HCC group. The pathological features for samples in the WCH-NonHBV-HCC is distinct, and these different pathological patterns include pseudoglandular histological pattern (A52), fibrolamellar-HCC (A19), trabecular histological pattern (A107) and steatosis (A66), etc.
