# Supplementary material for: Comprehensive analysis of genomic and immunological profiles in Chinese and Western hepatocellular carcinoma populations
Source: Aging (Albany NY). 2021 Apr 18;13(8):11564–94. doi: 10.18632/aging.202853 (PMC8109067; doi:10.18632/aging.202853)
Supplement: Supplementary Tables [file aging-13-202853-s003.pdf]

SUPPLEMENTARY TABLES

Supplementary Table 1. Detailed information of the primary antibodies.

Antibody	Source	Species	Dilution	Antigen retrieval
CD20	Abcam, ab9475	Mouse	1:200	Citrate buffer (pH 6.0) microwave 16min
CD3	Abcam, ab16669	Rabbit	1:200	Citrate buffer (pH 6.0) microwave 16min
CD8	Abcam, ab33786	Mouse	1:200	Citrate buffer (pH 6.0) microwave 16min

Supplementary Table 2. Clinicopathologic characteristics of WCH cohort according to HBV infection.

	WCH-NonHBV-HCC (N=19)	WCH-HBV-HCC (N=81)	P-value
Age, years	59.3 ± 10.7	49.7 ± 11.3	0.002
Gender			0.154
Male	14 (73.7%)	71 (87.7%)	
Female	5 (26.3%)	10 (12.3%)	
Alpha-fetoprotein, ng/mL			0.448
<400	13 (68.4%)	47 (58.0%)	
≥400	6 (31.6%)	34 (42.0%)	
Tumor size, cm	7.9 ± 4.9	6.7 ± 4.1	0.297
Tumor number			0.143
Single	9 (52.9%)	58 (73.4%)	
Multiple	8 (47.1%)	21 (26.6%)	
TNM stage			0.272
I	6 (31.6%)	34 (42.0%)	
II	7 (36.8%)	27 (33.3%)	
IIIA	5 (26.3%)	9 (11.1%)	
IIIB	1 (5.3%)	11 (13.6%)	
Tumor differentiation			1.000
High	8 (47.1%)	40 (50.6%)	
Low	9 (52.9%)	39 (49.4%)	
Microvascular invasion			1.000
No	13 (68.4%)	57 (70.4%)	
Yes	6 (31.6%)	24 (29.6%)	

Supplementary Table 3. Clinicopathologic characteristics of TCGA cohort.

	TCGA-Alcol-HCC (N=69)	TCGA-HBV-HCC (N=106)	P-value
Age, years	63.5 ± 11.6	54.8 ± 11.7	<0.001
Gender			0.734
Male	58 (84.1%)	87 (82.1%)	
Female	11 (15.9%)	19 (17.9%)	
Race			<0.001
White	41 (61.2%)	9 (8.7%)	
Asian	25 (37.3%)	92 (88.5%)	
Black	1 (1.5%)	3 (2.9%)	
Alpha-fetoprotein, ng/mL			0.518
<400	32 (80.0%)	74 (73.3%)	
≥400	8 (20.0%)	27 (26.7%)	
TNM stage			<0.001
I	23 (38.3%)	71 (68.3%)	
II	12 (20.0%)	21 (20.2%)	
III	25 (41.7%)	10 (9.6%)	
IV	0 (0.0%)	2 (1.9%)	
Tumor differentiation			<0.001
High	50 (72.5%)	44 (41.5%)	
Low	19 (27.5%)	62 (58.5%)	
Vascular invasion*			0.032
No	23 (53.5%)	74 (71.8%)	
Yes	20 (46.5%)	29 (28.2%)	

*Including macro- and micro-vascular invasion.
